# Physiological phenotypes have optimal values relevant to healthy aging: sweet spots deduced from the Canadian Longitudinal Study on Aging

**DOI:** 10.1007/s11357-023-00895-2

**Published:** 2023-09-09

**Authors:** Olga Vishnyakova, Xiaowei Song, Kenneth Rockwood, Lloyd T. Elliott, Angela Brooks-Wilson

**Affiliations:** 1 Canada’s Michael Smith Genome Sciences Centre, BC Cancer, Vancouver, BC Canada; 2 Department of Biomedical Physiology and Kinesiology, Simon Fraser University, Burnaby, BC Canada; 3 Department of Statistics & Actuarial Science, Simon Fraser University, Room SC K10545, 8888 University Drive, Burnaby, BC V5A 1S6 Canada; 4 Surrey Memorial Hospital, Fraser Health Authority, Surrey, BC Canada; 5 Division of Geriatric Medicine, Dalhousie University, Halifax, NS Canada

**Keywords:** CLSA, Healthy aging, Homeostasis, Sweet spots, Heteroskedasticity, Physiological measures

## Abstract

Previous observations on a group of exceptionally healthy “Super-Seniors” showed a lower variance of multiple physiological measures relevant for health than did a less healthy group of the same age. The finding was interpreted as the healthier individuals having physiological measurement values closer to an optimal level, or “sweet spot.” Here, we tested the generalizability of the sweet-spot hypothesis in a larger community sample, comparing differences in the variance between healthier and less healthy groups. We apply this method to the Canadian Longitudinal Study on Aging (CLSA) comprehensive cohort of 30,097 participants aged 45 to 85 years with deep phenotype data. Data from both sexes and four age ranges were analyzed. Five instruments were used to represent different aspects of health, physical, and cognitive functioning. We tested 231 phenotypic measures for lower variance in the most healthy vs. least healthy quartile of each sex and age group, as classified by the five instruments. Segmented regression was used to determine sex-specific optimal values. One hundred forty-two physiological measures (61%) showed lower variance in the healthiest than in the least healthy group, in at least one sex and age group. The difference in variance was most significant for hemoglobin A1c and was also significant for many body composition measurements, but not for bone mineral density. Ninety-four phenotypes showed a nonmonotonic relationship with health, consistent with the idea of a sweet spot; for these, we determined optimal values and 95% confidence intervals that were generally narrower than the ranges of current clinical reference intervals. These findings for sweet spot discovery validate the proposed approach for identifying traits important for healthy aging.

**Supplementary Information:**

The online version contains supplementary material available at 10.1007/s11357-023-00895-2.

## Introduction

Homeostasis is the collection of mechanisms that living organisms use to maintain physiological constancy by regulating their internal environment [1]. Homeostasis causes variables such as glucose level, body temperature, or blood pressure to be maintained close to optimal values that we will refer to as “sweet spots” [2]. Such regulation is usually associated with better health. For example, deviation from the optimal range for vitamin D, involved in calcium homeostasis, is associated with mortality risk [3, 4]; fasting glucose levels in an optimal range are associated with a lower risk of mortality and cardiovascular events [5]. Homeostatic imbalance, or homeostenosis, occurs when normal physiological control is disrupted. Such instability can result in disease. For example, dysregulation of lipid metabolism leads to dyslipidemia and kidney injury [6], and impaired glucose tolerance causes diabetes and raises the risk of cardiovascular diseases [7]. Homeostasis is a lens through which to view, analyze, and understand phenotypes that are important for, and can be indicators of health [8, 9].

Aging is a major factor in the disruption of homeostatic regulation, exemplified by the loss of proteostasis, increased inflammation, or compromised autophagy [8–10]. Manifestation, onset, and rate of aging processes are heterogeneous in older adults. Moreover, health heterogeneity tends to increase with age [11]. The US Census Bureau projects that one in five Americans will be 65 or over by 2030 [12]; this underlines the importance of understanding which measures are important for, or indicative of health, and the lifestyle and other factors associated with physiological dysregulation.

To characterize health or physiological dysregulation, several research groups have constructed composite indices incorporating laboratory measures or health histories. Allostatic load (AL) estimates physiological response to stress and is associated with an age-related reduction in cognitive and physical function [13]. Studies over the past two decades have estimated the AL index using a variety of biomarker combinations that reflect the states of the cardiovascular, metabolic, and immune systems [14]. The multivariate Mahalanobis distance from observed phenotypes’ means was proposed to examine longitudinal changes suggesting a positive correlation with age and subsequent mortality [15]. Multidimensional factors of positive aging have also been identified using principal components factor analysis [16]. However, despite strong association with age, risk of mortality, cognitive, and physical functioning, the aggregate nature of multivariate indices hampers interpretability, and illumination of the etiology and genetic basis of dysregulation and age-related changes. In contrast to previous studies focusing on constructing summary measures to access individuals’ health and forecast health trajectories and adverse health outcomes [16–19], we concentrate on a procedure that has a potential to disclose novel measures related to health and establish optimal ranges for specific phenotypes.

Studying healthy long-lived individuals allows us to identify unrecognized features of healthy aging. To that end, the Super-Seniors Study recruited healthy oldest-old people to study genetic factors associated with healthy aging [20]. The Super Seniors are healthy individuals aged 85–110 years who at recruitment had never been diagnosed with cancer (except non-melanoma skin cancer), cardiovascular disease, major pulmonary disease, dementia, or diabetes [20]. A key observation was that this cognitively and physically high-functioning group showed lower telomere length variance [21]. In contrast to studies that examine relationships between the telomere lengths of individuals and their health status [22], we focused on the difference in telomere length variance between a healthy group vs. a less healthy group. We seek to advance the hypothesis that in healthier groups, measures important for health tend to be closer to optimal values. This provides a way to identify traits important for health and likely to be homeostatically controlled. Understanding sweet spots and what affects them (and how to maintain proximity to sweet spots through lifestyle or medical intervention) has the potential to extend the healthspan of people and populations.

The objective of our study is to identify physiological measures previously lesser known to be relevant to healthy aging. We propose a statistical framework for detecting health-related phenotypes through examining the variance and determining sex-specific optimal values. We also explored the lifestyle and socioeconomic factors associated with deviation from estimated sweet spots by testing additional covariates.

## Methods

### Data source and participants

We examine data from the Canadian Longitudinal Study on Aging (CLSA) [23]. These data involve a stratified sample of 51,338 Canadian males and females aged 45 to 85 years. Community-dwelling participants were recruited from the ten Canadian provinces excluding individuals unable to respond in English or French; residents of the three Canadian territories; individuals living on First Nation reserves or in nursing homes; full-time members of the armed forces; and individuals with significant cognitive impairment [23]. We analyzed a subsample of 30,097 participants included in the comprehensive cohort (COM) of CLSA that underwent a comprehensive physical assessment including blood and urine specimens.

### Instruments for the classification of health status

For this study, five instruments were applied, representing health status in different ways. Table 1 provides a list and characteristics of the assessment tools (i.e., instruments) used. To allow comparability between instruments, all estimators were transformed into the unit interval with the upper boundary for health deficits representing the least healthy individuals.

**Table 1:** Health instruments and their characteristics

Instrument	Description	Characteristics (mean; SD)	
		Females	Males
I	The frailty index as a normalized sum of 51 health deficits	0.092; 0.061	0.081; 0.055
II	The normalized number of chronic conditions: cancer (except non-melanoma skin cancer), cardiovascular disease, major pulmonary disease, dementia, and diabetes	0.126; 0.152	0.155; 0.158
III	The normalized number of 28 self-reported clinician-diagnosed conditions (except conditions used for Instrument II)	0.121; 0.089	0.082; 0.067
IV	Composite cognitive score as the mean of three cognitive domain scores (memory, the executive functions, the psychomotor speed domains) derived from rank normalizes scores of domain-specific tests*	0.445; 0.180	0.475; 0.185
V	Physical functioning as a mean rank normalizes scores of five physical assessment tests**	0.498; 0.185	0.419; 0.177

^*^The Rey Auditory Verbal Learning Test–Trial 1 within the memory domain [24]; the Mental Alternation Test [25], animal fluency 1 Test [26], Controlled Oral Word Association Test [27], Stroop Test [28, 29] and Prospective Memory Test [30] within the executive functions domain; and choice reaction times [31] within the psychomotor speed domain

^**^Timed (4-m) walk test, timed get up and go test, standing balance test, chair rise test, and hand grip strength

To construct Instrument I, a deficit accumulation frailty index (FI), we utilized 51 CLSA measures that meet the standard criteria [32, 33]. Instrument II was defined as the normalized number of medical conditions that were exclusion criteria for Super-Seniors: cancer (except non-melanoma skin cancer), cardiovascular disease, major pulmonary disease, diabetes, and dementia. Participants taking medication for diabetes or heart disease or undergoing treatment for any of these diseases were also considered to have a confirmed disease status. Instrument III was a count of 28 self-reported clinician-diagnosed chronic conditions excluding those used to define Instrument II. Instrument III includes phenotypes that are not associated with age, such as asthma, epilepsy, anxiety disorder, migraine, headaches, and traumatic brain injury; and age-related deficits used in the FI.

We also constructed two separate continuous instruments reflecting cognitive (Instrument IV) and physical (Instrument V) functioning based on neuropsychological and mobility assessment tests. As a preprocessing step for these two instruments, we determined the direction of risk for each assessment test score and inverted values if necessary to make test results increase with cognitive or physical decline. To assess cognitive capacity, we constructed a composite cognitive score. CLSA included assessments in three cognitive domains as an alternative to global cognitive screening measures [34]. To calculate composite cognitive scores, some studies have used the mean of cognitive domain *z*-scores derived from means of domain-specific tests [35]. In contrast, we applied rank normalization to preserve identical ranges for all instruments used for experiments, to allow for comparison across instruments [36]. We constructed a composite cognitive score using seven tests: the Rey Auditory Verbal Learning Test–Trial 1 within the memory domain [24]; the Mental Alternation Test [25], animal fluency 1 test [26], Controlled Oral Word Association Test [27], Stroop Test [28, 29], and Prospective Memory Test [30] within the executive functions domain; and choice reaction times [31] within the psychomotor speed domain. For Instrument V, physical function, we used a mean of rank normalized scores of the following assessments: timed (4-m) walk test, timed get-up-and-go test, standing balance test, chair rise test, and hand grip strength. Lists of measures used for each instrument, directions of risk, and methods of operationalization can be found in the Supplemental Materials (Supplemental Table S1).

### Phenotypes

Phenotypes included 317 traits from the following categories: hematology; blood chemistry; electrocardiogram (ECG); body measures including BMI, hip, and waist circumferences; bone mineral density and body composition data collecting using dual energy X-ray scanners; vital signs; and inflammatory biomarkers (Supplementary Table S2).

### Covariates

Covariates included age group at baseline (stratified according to the levels < 55, 55–64, 65–74, and ≥ 75 years), self-reported sex, self-reported ancestry, alcohol consumption (never, light/moderate, heavy drinker), household income (CDN < $20,000, $20,000–$50,000, $50,000–$100,000, $100,000–$150,000, > $150,000), education level (< secondary, secondary, post-secondary diploma/certification, or university degree), nutritional risk (high/low), smoking status (never, occasional, current smoker, current heavy smoker), province of residence, living area (urban, rural), retirement status, physical activity level, and living alone. In CLSA, nutritional assessment was performed by the AB SCREEN II [37] and physical activity level was assessed by the Physical Activity Scale for Elderly [38]. High nutrition risk was identified as a score below a validated cutoff of 38 [39]. We categorized self-reported ancestry into Arab, Black, Asian, Latino, White, or other.

### Statistical analysis

Using the five instruments, we calculated health deficit scores for the CLSA COM participants. For continuous instruments (I, III, IV, and V), we assigned health levels according to quartiles: healthiest, good health, fair health, and least healthy. Cutoffs were applied to categorize participants by health levels regardless of age and sex while controlling for both these factors in the analysis. For Instrument II, participants without any of the five major disorders (cancer, cardiovascular disease, major pulmonary disease, diabetes, and dementia) were considered the healthiest group, whereas individuals diagnosed with 2 or more major disorders were assigned to the least healthy group. To avoid confounding by ethnicity, we restricted the primary analysis to the largest group which was white.

The sweet spot discovery pipeline has two main steps: (1) identify phenotypes that are relevant for health by testing for the variance effect; and (2) estimate the position of sweet spots. To examine phenotypes for associations of variance with health, a Brown-Forsythe (BF) test for heteroskedasticity was utilized due to skewness and unequal sample size [40]. For each 10-year age bin and self-reported sex, the equality of phenotype variances was pairwise tested between the healthiest and least healthy groups. According to the hypothesis, we expect to observe significantly lower variance in the healthiest group for phenotypes related to health. Analysis was restricted to complete cases (participants without missing values for any phenotype used to calculate deficit score or for a phenotype of interest) with at least 30 individuals within each group. Measures for which the healthiest and least healthy groups had significantly different variance for at least one age and sex group, and for which the healthiest group had the lower variance were selected for further examination. Bonferroni adjustment was used to account for multiple testing.

We characterized the relationship between each instrument and phenotype magnitude to identify the type of relationship and sweet spot value by examining breakpoints using segmented regression where the effect on the health deficit score changed [41–44]. This approach is effective in determining the value of a measure where the effect on the health deficit score changed and resulted in meaningful regression parameters. We applied a model with a single-free breakpoint assuming that a continuous phenotype affects the mean response of health deficit scores via two straight lines that are connected at a breakpoint. Breakpoints are identified as optimal values, e.g., sweet spots, under the following conditions: (1) slopes of the straight lines have different signs; (2) 95% confidence intervals for both slopes do not contain zeros; and (3) the difference in slope is significant, tested using a two-sided score test. We also assumed that the positions of sweet spots are fixed regardless of age but not sex. To avoid confounding by age, we regressed health deficit scores onto age and used the residuals for the segmented regression model. Analysis for males and females was done separately to determine sex-specific estimates of sweet spots. Results were adjusted for multiple testing using Bonferroni correction and adjusted *P* values less than 0.05 were considered statistically significant.

To explore the effect of lifestyle and socioeconomic factors, health instruments were regressed onto the covariates, and the residuals from these were used for regression analyses. We interpret a change in a pattern of interaction between a health deficit score and measures as evidence of an impact of these factors on a phenotype’s values. This implies the potential usefulness of the intervention to approach the optimal phenotypic value. All tests were performed in the R software, version 3.6, without sampling weights.

### Replication

Given the substantially smaller sample size of other ethnicity groups in CLSA, the replication of the segmented regression results was performed only for the subset of phenotypes and groups for which there was sufficient statistical power. Similar to the primary analysis, the replication was performed separately for females and males. For a segmented regression model with one free breakpoint, power calculation is based on the score statistic [45] implemented in the R package *segmented* (v1.6–4) [46]. We used following assumptions for power analysis: (1) the sample size for each group was assumed to be given by the mean sample size across phenotypes and sexes; (2) the covariates were assumed to be normally distributed; (3) the estimate for the breakpoint value was the mean value of the phenotype; (4) the effect sizes for phenotypes do not vary greatly between groups. Due to the novelty of the analysis, effect sizes for phenotypes derived from the difference in slopes cannot be established from existing literature. Hence, we used values for slope differences for each phenotypic model from the primary analysis on white group. In addition, for the power analysis, the type I error probability was set to 0.05 and the response standard deviation was set to the mean standard deviation across health instruments. The subset of phenotypes for replication was selected based on the slope difference required to reach the desired power level of 0.8.

## Results

### Participant measures and characteristics of health scores

Of 30,097 individuals from the CLSA COM, 52.3% were female, and the average age was 63.1 (95%CI: 63.0–63.2) years. Ninety-four percent of participants self-reported being white; the second largest ethnicity group was Asian with 2% of the CLSA COM cohort; less than 0.7% of CLSA adults identified themselves as belonging to the third largest ethnic group, which was black.

The distributions for health instruments the FI (I), five major diseases (II), and other chronic conditions (III) were skewed to the right, while rank normalization used to assess cognitive and physical functioning in instruments IV and V led to normally distributed scores. The strongest correlation between health scores was observed for the FI (I) and other chronic conditions (III) (*r*^2^ = 0.68), due to the partial intersection of phenotypes. All health deficit scores were higher for participants in the oldest age group (> 75 years). Across all instruments, mean health deficit scores were strongly correlated with age (*r* > 0.90). Across all ages, males had higher mean scores than females for five major diseases (II) and cognitive functioning (IV) while lower for other instruments consistent with previous studies [47, 48]. Supplementary Fig. S1 (A–E) shows distributions for all health instruments and association between mean scores and age.

Among all CLSA measures within the eight categories considered in the analysis, 231 were selected for sweet spots testing after exclusion of highly correlated variables (*r* > 0.9) and measures with more than 24% missing values except for inflammatory biomarkers. TNF-alpha and IL-6 measures are available only for a third of CLSA COM participants. Considering the reported importance of these biomarkers for frailty, we included these two measures even though they were above the missingness threshold, and performed testing for the subset of participants with available data. Instead of imputing missing phenotypic values, we restricted our analysis to complete cases. The sample size varies based on the model and the phenotype (missingness varies by phenotype). The sample sizes in each analytical model for the subsets of participants self-reporting as white ranged from 4595 to 14,524 (median 14,524) for females and from 4411 to 13,848 (median 13,259) for males. For the Asian subset, the second largest ethnic group, the sample size ranged from 87 to 268 (median 260) for females and from 117 to 360 (median 349) for males. For the black ethnic group, the sample size ranged from 28 to 111 (median 105) for females and from 26 to 110 (median 104) for males. Detailed information is shown in Supplementary Table S3.

### Health-related phenotype discoveries

Using the instruments, we assigned health levels to each participant. The first, second, and third quartiles of the FI (I) were 0.04, 0.07, and 0.11, respectively. Thus, individuals with the FI < 0.04 were considered to be the healthiest group, whereas those with score above 0.11 were considered to be the least healthy group. Similarly, we assign these two health levels for other continuous instruments, other chronic conditions (III), cognitive (IV), and physical functioning (V) using cutoffs (< 0.04, > 0.14), (< 0.35, > 0.61), and (< 0.33, > 0.6) for the healthiest and the least healthy groups, respectively.

**Fig. 1:**
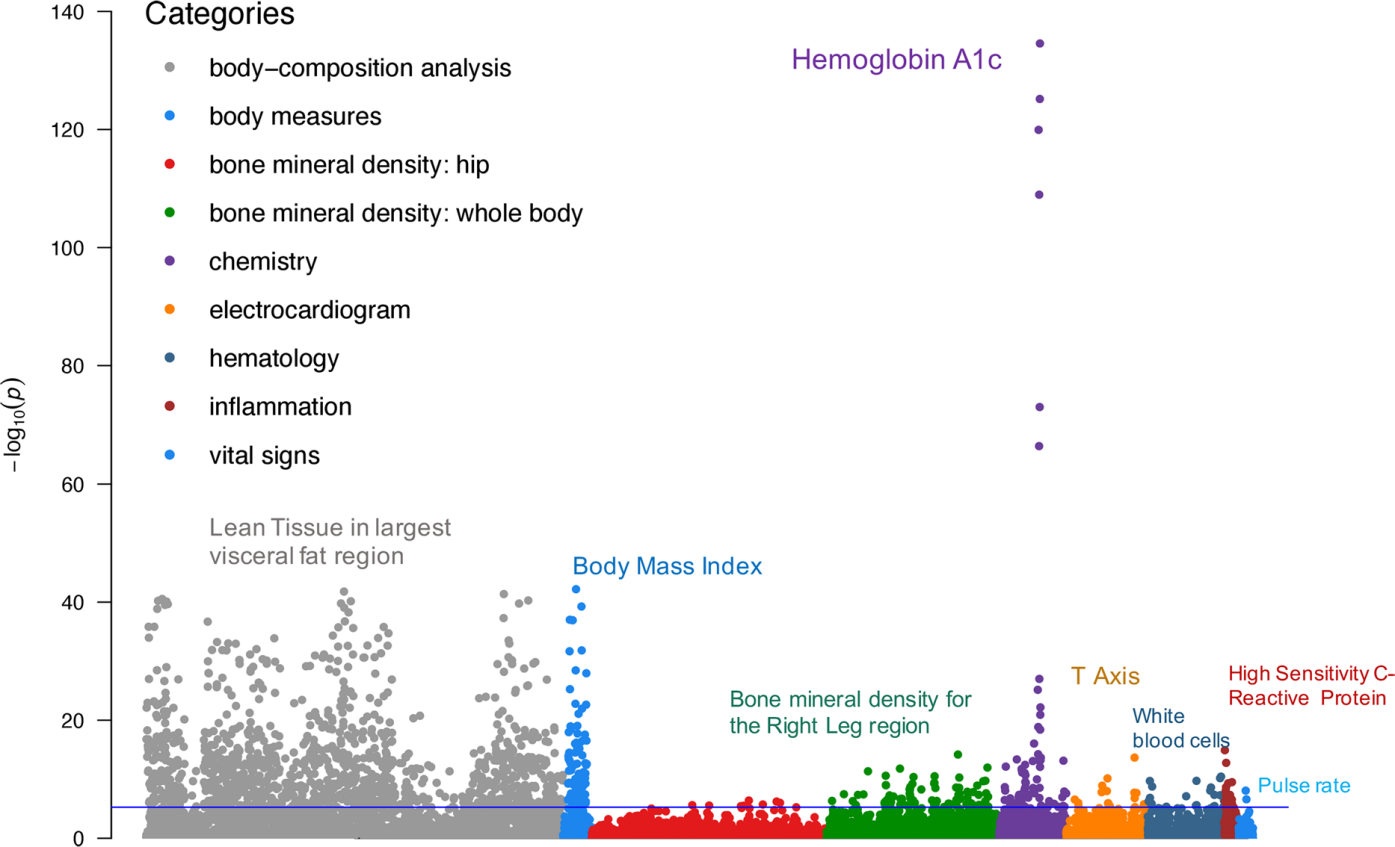
Brown-Forsythe test results for all instruments and groups for all phenotypes. The *x*-axis indicates phenotypes ordered by categories and labels; the *y*-axis indicates log-transformed *P*-values

To identify phenotypes related to health and homeostasis, we tested for homogeneity of variance between the most and least healthy groups, for each phenotype-instrument pair, age category, and sex, which resulted in 9240 tests (#measures × #instruments × #groups). Overall, the BF test detected 142 physiological measures with significantly lower variance for the healthiest, compared to the least healthy adults, in at least one sex and age group after Bonferroni correction. Figure 1 displays an overall summary of results for all measures, categories, instruments, and groups. The most significant difference in variance was for the phenotype hemoglobin A1c (HbA1c), a diagnostic criterion for diabetes. This phenotype showed heteroskedasticity for all instruments except other chronic conditions (III) (*P* = 0.16). Moreover, five major diseases (II) test results for hemoglobin A1c variance were consistently significant across all age groups and sexes (*P* < 0.001). The second most significant measure was body mass index (BMI), which showed variance heterogeneity for both sexes by all instruments (*P* < 0.001) except for cognitive function (IV).

Among body composition phenotypes derived from images collected using Hologic QDR 4500 dual energy X-ray scanners, 91% revealed heteroskedasticity. In contrast, only 16% of bone mineral density phenotypes showed variance heterogeneity. By body part, variance between the estimated fat and lean content of visceral, android, and gynoid regions was much larger for the least healthy than for the healthiest individuals. Significant results for ECG traits were detected using instruments the FI (I), five major diseases (II), and physical functioning (V) across all age groups. T-axis, QRS duration, Q onset, and P offset revealed heteroskedasticity for both sexes while P duration showed significant difference in variance among 55–65-year-old men. Variance for measures of vital signs was marginally higher in the least healthy group, but only pulse rate remained significant with multiple testing corrections. Similarly, only Interleukin-6 and high-sensitivity C-reactive protein showed variance heterogeneity in the inflammatory biomarkers category (*P* < 0.001). We detected heteroskedasticity for all available blood chemistry measures with the exception of 25-hydroxyvitamin D and six of 16 hematology measures, after Bonferroni adjustment. Because levels of 25-hydroxyvitamin D of less than 12 nmol/L or greater than 150 nmol/L are associated with higher mortality risk in the literature ^3,4^, our expectation was that this phenotype would show a sweet spot; however, analysis revealed equality of variances across all age groups. Season of blood draw had no effect on vitamin D level, *P* > 0.05.

Although heteroskedasticity was observed across all instruments, the number of phenotypes detected per instrument varied. Using the instruments the FI (I), five major diseases (II), other chronic conditions (III), and physical functioning (V), we observed significantly lower variance among the healthiest adults for 105, 113, 87, and 115 measures, respectively. Supplementary Fig. S2-A and Table S4 show overlapping results between instruments. Only two phenotypes showed unequal variance between health groups determined by cognitive functioning (IV), cognitive function: hemoglobin A1c (*P* = 0.02) and percentage of fat tissue to total tissue mass in the head region (*P* = 0.002).

Overall, we observed heteroscedasticity for 95 measures in both sexes. As for the rest, 29 health-related phenotypes were detected only in females, including cholesterol, ferritin, and triglycerides, and 18 were detected only in males, including hemoglobin (Supplementary Fig. S2-B and Table S5). Detailed results stratified by age group, sex, and instrument are presented in Table 2. Physiological measures that revealed variance heterogeneity for at least one instrument were used for relationship analysis.

**Table 2:** Variance heterogeneity between the least and most healthy groups detected in self-reported white CLSA participants

Phenotype*	Instrument**	Sex	*P*-value***
Blood chemistry			
Hemoglobin A1c, %	I, II, IV, V	M, F	$2.1\times {10}^{-131}$
Free thyroxine, pmol/L	I, III	M, F	$7.2\times {10}^{-13}$
Cholesterol, mmol/L	I, II, V	M, F	$3.5\times {10}^{-10}$
Triglycerides, mmol/L	I, II, III, V	F	$5.8\times {10}^{-10}$
Estimated glomerular filtration rate, mL/min/1.73m^2^	I, II	M, F	$2.9\times {10}^{-9}$
Alanine aminotransferase, U/L	I, II	M, F	$5.8\times {10}^{-9}$
Creatinine, µmol/L	I, II, V	M, F	$1.4\times {10}^{-8}$
Ferritin, µg/L	I	F	0.001
Albumin, g/L	II	M, F	0.001
Hematology			
White blood cells, 10^9^/L	I, II, V	M, F	$2.7\times {10}^{-7}$
Monocytes, 10^9^/L	I, II	F	$1.4\times {10}^{-6}$
Granulocytes, 10^9^/L	I, II, V	M, F	$1.5\times {10}^{-6}$
Red blood cell distribution width, %	I, II, V	M, F	$1.9\times {10}^{-5}$
Mean corpuscular volume, fL	V	F	0.0005
Hemoglobin, g/L	V	M	0.03
Inflammatory biomarkers			
High sensitivity C-reactive protein, mg/L	I, II, III, V	M, F	$9.3\times {10}^{-12}$
Interleukin-6 (IL-6), pg/mL	I, II, V	M, F	$2.4\times {10}^{-6}$
Electrocardiogram			
T-axis, degree	I, II, V	M, F	$1.7\times {10}^{-10}$
QRS duration, ms	II, V	M, F	$5.5\times {10}^{-7}$
Q onset, ms	II	M, F	$9.5\times {10}^{-6}$
P duration, ms	II	M	0.002
P offset, ms	II	M, F	0.007
Body measures			
Body mass index	I, II, III, V	M, F	$5.5\times {10}^{-39}$
Hips circumference, cm	I, II, III, V	M, F	$5.5\times {10}^{-36}$
Average weight, kg	I, II, III, V	M, F	$8.2\times {10}^{-34}$
Waist circumference, cm	I, II, III, V	M, F	$9.6\times {10}^{-25}$
Body-composition analysis			
Lean tissue in largest visceral fat region, g	I, II, III, V	M, F	$1.5\times {10}^{-38}$
Lean tissue in android region, g	I, II, III, V	M, F	$4.3\times {10}^{-31}$
Fat tissue in trunk region, g	I, II, III, V	M, F	$1.3\times {10}^{-30}$
Total fat mass divided by height squared, kg/m^2^	I, II, III, V	M, F	$2.7\times {10}^{-30}$
Fat tissue in gynoid region, g	I, II, III, V	M, F	$1.2\times {10}^{-28}$
Bone mineral density			
Bone mineral density for the right leg region	I, II, III, V	M, F	$5.1\times {10}^{-11}$
Total bone mineral density excluding the head region	I, II, III, V	M, F	$8.3\times {10}^{-9}$
Bone mineral content for the pelvic region	II, III, V	F	$1.2\times {10}^{-8}$
Bone area for the right rib region	V	M	$3.5\times {10}^{8}$
Vital signs			
Average pulse rate (excluding 1st reading)	II, V	F	$6.5\times {10}^{-5}$

^*^Analysis was stratified by sex. The final sample sizes in each analytical model range from 4595 to 14,524 (median 14,524) for females and from 4411 to 13,848 (median 13259) for males

^**^Instruments: I — the frailty index; II — the number of five diseases: cancer (except non-melanoma skin cancer), cardiovascular disease, major pulmonary disease, dementia, and diabetes; III — the number of other chronic conditions; IV — composite cognitive score; and V — physical functioning. These are described in Table 1

^***^Comparison of least and most healthy groups variance based on the Brown-Forsythe test. *P*-values were adjusted for 9240 tests using the Bonferroni correction. Only the lowest *P*-value across 40 tests (5 instruments × 2 sexes × 4 age groups) for each phenotype is reported in a table. Only the most significant results from each category are presented in this table

### Relationship investigation

**Fig. 2:**
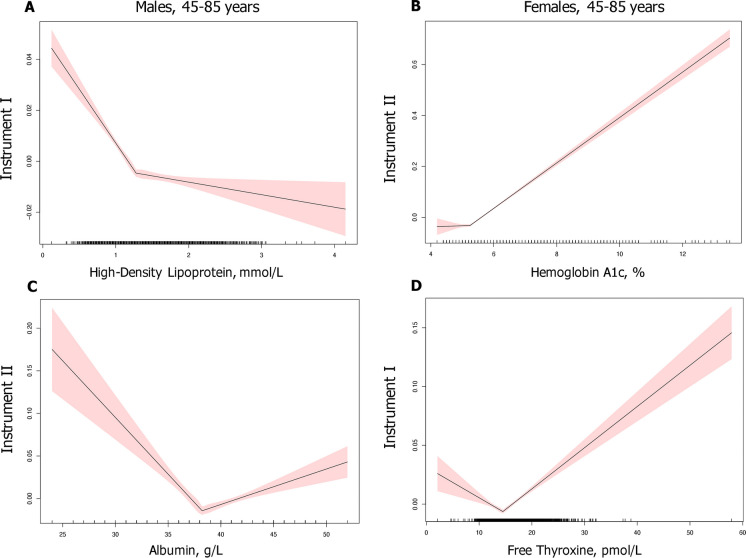
Types of segmented relationships:** A** Decreasing for high-density lipoprotein; **B** increasing for HbA1c; **C** nonmonotonic for albumin with sweet spot estimated at 38.3 g/L (95%CI [37.7, 38.8]) in males using Instrument II; and **D** nonmonotonic for free thyroxine with sweet spot estimated at 14.6 pmol/L (95%CI [14.1, 15.1]) in females using Instrument I. Instruments: I — the frailty index; II — the number of five diseases: cancer (except non-melanoma skin cancer), cardiovascular disease, major pulmonary disease, dementia, and diabetes

Using segmented regression with a single breakpoint, we determined three main types of segmented relationships assuming an absence of discontinuities between segments: increasing, decreasing, and nonmonotonic (decreased for phenotype values less than the breakpoint and increased otherwise) (Fig. 2). For the 142 variables examined, we detected 83 positive and 33 negative associations between measures and health deficit. For the 94 measures that revealed a nonmonotonic relationship, we determined optimal values along with 95%CI at breakpoints by testing for a non-zero difference in slope parameter and requiring slopes to have opposite signs. Results were adjusted for 1420 tests (#measures × #sexes × #instruments). A summary of the results organized by categories and instruments is shown in Table 3.

**Table 3:** Breakpoints estimates using segmented regression analysis on phenotypes detected in self-reported white CLSA participants

Phenotype*	Instrument**	Sex	Breakpoint	95%CI	*P*-value***
Blood chemistry					
Free thyroxine, pmol/L	I	M	14.6	[13.8, 15.4]	0.002
	I	F	14.6	[14.1, 15.1]	0.002
Low-density lipoprotein, mmol/L	IV	F	2.5	[2.3, 2.7]	$1.5\times {10}^{-5}$
Estimated glomerular filtration rate, mL/min/1.73m^2^	I	M	75	[73, 77]	$3.6\times {10}^{-50}$
	I	F	70	[67, 72]	$2.3\times {10}^{-32}$
Alanine aminotransferase, U/L	II	M	9.7	[8.4, 11.1]	$5.9\times {10}^{-4}$
	I	F	9.7	[8.6, 10.8]	0.003
Creatinine, µmol/L	II	F	70.8	[68.6, 72.0]	$1.7\times {10}^{-4}$
	V	F	69.9	[67.9, 71.9]	0.02
Ferritin, µg/L	I	M	262	[210, 313]	$5.0\times {10}^{-5}$
Albumin, g/L	II	M	38.3	[37.7, 38.8]	$2.2\times {10}^{-14}$
	IV	M	38.7	[37.6, 39.8]	$1.6\times {10}^{-4}$
Hematology					
Mean corpuscular volume, fL	V	M	95.2	[93.9, 96.4]	0.007
	V	F	93.3	[92.16, 94.4]	$3.4\times {10}^{-6}$
Hemoglobin, g/L	V	M	130	[127, 133]	$8.5\times {10}^{-5}$
	II	F	149	[145, 153]	$5.8\times {10}^{-6}$
Electrocardiogram					
*T*-axis, degree	II	M	46	[39, 51]	$3.6\times {10}^{-10}$
	II	F	52	[45, 57]	$6.0\times {10}^{-7}$
QRS duration, ms	V	M	84.74	[80.7, 88.8]	0.015
	V	F	91.8	[86.8, 96.6]	$1.7\times {10}^{-4}$
Q onset, ms	V	F	438	[435, 441]	0.01
P duration, ms	I	M	109.3	[104.5, 114.1]	$8.7\times {10}^{-4}$
	I	F	91.6	[85.5, 97.7]	$7.8\times {10}^{-5}$
Body measures					
Body mass index	V	M	24.2	[23.7, 24.7]	$8.4\times {10}^{-10}$
Average weight, kg	I	M	73.5	[70.8, 76.2]	$4.8\times {10}^{-5}$
	I	F	58.4	[56.5, 61.0]	$6.7\times {10}^{-4}$
Body-composition analysis					
Lean tissue in largest visceral fat region, g	V	M	2306	[2244, 2368]	$1.0\times {10}^{-18}$
Lean tissue in android region, g	V	M	4412	[4272, 4553]	$1.4\times {10}^{-17}$
Lean mass in limbs divided by height squared, kg/m^2^	V	M	8.4	[8.2, 8.5]	$1.8\times {10}^{-43}$
	V	F	6.9	[6.8, 7.0]	$5.1\times {10}^{-39}$
Bone mineral density					
Bone mineral density for the right leg region	V	M	1.39	[1.36, 1.42]	$1.3\times {10}^{-25}$
	V	F	1.19	[1.15, 1.23]	$1.5\times {10}^{-12}$
Total bone mineral density excluding the head region	V	F	1.03	[1.00, 1.07]	$1.1\times {10}^{-9}$
Vital signs					
Average pulse rate (excluding 1st reading)	I	F	71	[68, 75]	$8.8\times {10}^{-5}$

^*^Analysis was stratified by sex. The final sample sizes in each analytical model range from 4595 to 14,524 (median 14,524) for females and from 4411 to 13,848 (median 13,259) for males. Health instruments were regressed against age. Residuals were used in the segmented regression model as outcome, phenotypic values as a covariate

^**^Instruments: I — the frailty index; II — the number of five diseases: cancer (except non-melanoma skin cancer), cardiovascular disease, major pulmonary disease, dementia, and diabetes; III — the number of other chronic conditions; IV — composite cognitive score; V — physical functioning. These are described in Table 1

^***^A non-zero difference-in-slope parameter of a segmented relationship is tested using a two-sided score test for a single change in the slope. *P*-values were adjusted for 1420 tests using the Bonferroni correction (5 instruments × 2 sexes × 142 phenotypes)

Regression analysis showed diverging associations with health instruments across phenotype categories. As expected, the variance of inflammatory biomarkers showed a positive association with the level of health deficits as assessed by all instruments for both sexes. For vital signs measures, average pulse rate showed a positive relationship with health deficit for older males. Conversely, we observed a nonmonotonic relationship between average pulse rate and instruments the FI (I), five major diseases (II), and physical functioning (V) for females. The estimated 95%CI were [67,75], [67,73], and [63,69], respectively. For body measures, waist, and hips circumference increased with health deficit measured by the FI (I) and five major diseases (II) for both sexes. Types of relationships for BMI varied by sex and instruments. For instance, we detected a breakpoint for males using physical functioning (V), 95%CI [23,25]. However, BMI increased with health decline for other instruments and for older females. For ECG measures, our segmented regression model estimated breakpoints for 5 out of 7 traits. For example, for T-axis, we found a nonmonotonic relationship across both sexes for instruments the FI (I), five major diseases (II), and physical functioning (V). Breakpoints for the model with the five major diseases were estimated at 46 degrees (95%CI: 40, 52) for males and 52 degrees (95%CI: 46, 58) for females. Health deficit increased with a decline in bone mineral content and lean mass. For blood chemistry measures, phenotypes showed diverging associations with different instruments. We observed a positive association between HbA1c and all instruments for both sexes. However, after exclusion of participants with either type1 or type 2 diabetes, a post hoc analysis provided an estimation of an optimal HbA1c level at 4.9% (95%CI [4.8, 5.1], *P* = 0.02) for older males using the FI (I). Aspartate aminotransferase (AST), creatinine, estimated glomerular filtration rate (eGFR), ferritin, albumin, and free thyroxine showed non-linear relationships. For example, we observed breakpoints for AST at 9.7 U/L (95%CI [8.4, 11.1]) and 9.7 U/L (95%CI [8.6, 10.8]) for males using five major diseases (II) and the FI (I), respectively.

Interestingly, for low-density lipoprotein (LDL), we detected optimal values for females at 2.6 mmol/L (95%CI [2.3, 2.9]) using cognitive functioning (IV) and at 2.5 mmol/L (95%CI [2.3, 2.7]) using physical functioning (V). In contrast, only negative associations were identified for males in models with instruments the FI (I), five major diseases (II), or physical functioning (V). To investigate whether medicine that lowers LDL altered the relationship type, we scanned self-reported medication data for CLSA COM participants and identified 7789 older adults taking statins, PCSK9 inhibitors, niacin, fibrates, and ATP citrate lyase (ACL) inhibitors. However, restricting regression analysis to participants who did not report using any of these medications revealed similar relationships.

We detected breakpoints for only two out of six hematology phenotypes that revealed heteroskedasticity. Particularly, optimal points for mean corpuscular volume were estimated at 93.3 fL (95%CI [92.16, 94.4]) for females and at 95.2 fL (95%CI [93.9, 96.4]) for males using physical functioning (V). Our estimates for hemoglobin were 130 g/L (95%CI [127, 133]) for older females using five major diseases (II) and 149 g/L (95%CI [145, 153]) for older males using physical functioning (V).

Stratified by instrument, the most optimal values were detected using the FI (I). Specifically, we identified 125 breakpoints for 73 measures across all categories except inflammatory biomarkers. For five major diseases (II), other chronic conditions (III), cognitive (IV), and physical (V) functioning, the segmented regression model revealed 20, 55, 14, and 124 breakpoints, respectively.

### Replication of sweet spots across ethnicities

We sought to repeat sweet spot estimation in ethnic groups other than white. Due to substantially smaller sample sizes, we calculated power and identified a subset of phenotypes to test for replication. We used the following parameters derived from the primary analysis on the white group: (1) mean sweet spot estimate was −0.24 (Fig. S3-A); (2) the health instrument’s standard deviation was 0.07; (3) the slope differences across phenotypes that showed optimal values ranged from 0.008 to 0.605 with mean 0.034 (Fig. S3-B). The mean sample sizes across phenotypes and sexes for the Asian and black groups were 282 and 95, respectively. For each difference in slopes within the range, we calculated power to detect breakpoints with a type-I error probability of 0.05. Figure S4 shows obtained power vs. slope difference for a given sample size. The power calculation revealed that replication could be done for phenotypes with a difference in slopes greater than 0.042 (62 phenotypes) for Asian and 0.068 (20 phenotypes) for black groups.

We repeated segmented regression on adults of Asian descent to estimate the sweet spot position for 62 phenotypes. After multiple testing correction for 620 tests (62 phenotypes × 2 sexes × 5 instruments), only low-density lipoprotein showed a nonmonotonic pattern with a significant difference in slopes. The optimal value was detected for males at 3.6 mmol/L (95%CI [3.2, 4.0]) using cognitive functioning (IV). Replication in the black ethnic group did not detect sweet spots for selected 20 phenotypes after correction for multiple testing.

In addition, we replicated results for HbA1c on nondiabetic Asian older adults. Particular interest in this measure is explained by the exceptional heteroskedasticity indicating high relevance to health. After exclusion of participants with diabetes, segmented regression analysis was performed for 180 females and 217 males of Asian descent using the FI (I). Similar to our primary analysis on the white group, we detected a breakpoint only for older nondiabetic males at 5.5% (95%CI [5.2, 5.8], *P* = 0.04). The observed difference in HbA1c level is consistent with a previous study [49].

### Impact of lifestyle and socioeconomic factors

To explore how sweet spots may be modulated by the environment, we regressed health instruments onto lifestyle and socioeconomic factors. We then performed segmented regression analysis on the residuals of the instruments. The CI intervals in the detected breakpoints and a nonmonotonic relationship were identified for 52 of 94 measures. The correlation between sweet spots estimates by both models was ${r}^{2}$ = 0.85.

## Discussion

This work presents a novel approach to detecting optimal values, or sweet spots, for physiological measures. We sought to cover two main gaps in the literature. First, we established optimal values by analyzing associations with health deficits rather than all-cause mortality. This approach is more sensitive and allows us to detect dysregulation for particular phenotypes at early stages. Concentrating on severe health outcomes may result in ranges outside of which damage is irreversible. Second, we applied the sweet spot discovery framework to the phenotypes across multiple domains using a large and well-characterized cohort of older adults where health deficits are easy to track. While previous studies have reported reference intervals for some physiological measures, e.g., BMI [50] or T-axis [51], optimal ranges are unknown for many other phenotypes, including body composition measures.

Not all measures collected by observational studies like CLSA are expected to be tightly regulated by the body and exhibit sweet spots; this is especially true when applied to variables derived from medical images, e.g., dual-energy X-ray absorptiometry (DXA). In our study, as a first step in the sweet spot discovery pipeline, we elucidated phenotypes likely to be regulated through analysis of variance heterogeneity. While the vast majority of studies of aging are focused on between-group differences in means, phenotypic heterogeneity remains neglected [52]. Our examination of variance in individual phenotypes suggests that many phenotypes are related to health and healthy aging. Assuming that these measures are likely to be homeostatically controlled and a deviation in either direction from their sweet spots leads to adverse health outcomes, we estimated optimal values by testing for a nonmonotonic relationship between the phenotype and health deficit scores.

In terms of confounders, sex and age are two main covariates that must be incorporated into the models. Sex is an important factor to consider because the optimal values of many biomarkers are already known to vary between males and females [53–55]. In our work, we approached this by stratifying by sex. This allowed us to discover new sweet spots that were sex-specific, and also determine which sweet spots were the same in men and women. With regard to age, the outcomes we consider are the instruments measures of health. As health and homeostasis tend to deteriorate with age [9, 10], age is strongly correlated with our instruments. Thus, age is an important factor to consider in our analysis. Optimal values themselves ideally should not depend on age for most of phenotypes. Although we observe changes in the level for some physiological measures, that does not imply the shift of optimal values. For instance, creatinine level decreases with age. However, it is caused by age-related decline in kidney function. While a value may deviate from a sweet spot due to age, our definition of sweet spot assumes that the optimal value is constant, and our test determines if deviation from it is associated with poor health. Thus, we have considered age in two ways. For the variance analysis (Table 2), we stratified our datasets by age group (splitting age into four levels), and for the segmented regression analysis (Table 3), we adjusted for age (by treating it as a confounder, and examining residuals after regression against age).

Heteroskedasticity between the least healthy and most healthy groups was observed for 61% of 231 phenotypes tested. The differences in the measurement value variance were observed across the phenotype categories, sexes, and age groups. HbA1c showed the most significant heteroskedasticity, consistent for all instruments, sexes, and age groups. Observations varied by how health is defined; physical functioning revealed a higher variance with greater health deficit, for the most phenotypes (*n* = 115) and the cognitive score the least (*n* = 2).

**Fig. 3:**
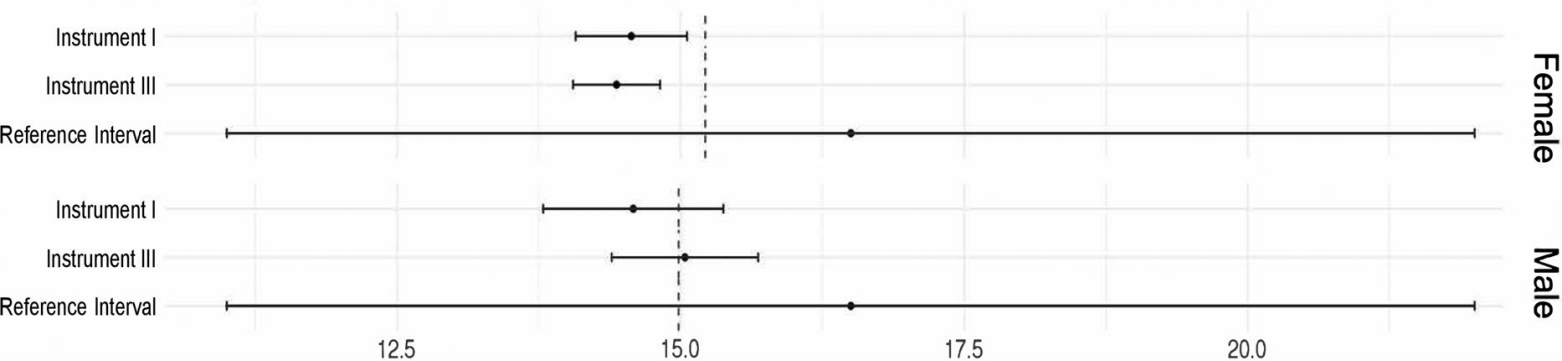
Optimal values with CI and reference intervals for free thyroxine. Dashed lines indicate mean values for females and males. Instrument I is the frailty index; Instrument III is the number of other chronic conditions. Reference intervals were adopted from textbook of *Clinical Chemistry and Molecular Diagnostics 5th Edition* [56]

A nonmonotonic relationship was seen for a subset (66%) of the phenotypes tested (*n* = 142), revealing optimal levels of the measurements in community-dewing adults 45 years and older. The hematology and blood chemistry data with known clinical reference intervals (such as hemoglobin, aspartate aminotransferase, creatinine or free thyroxine levels) showed the breakpoint 95%CI interval estimation within the standardized clinical threshold but of a narrower range (Fig. 3). The majority of sweet spots were identified by the FI, that includes deficits covering multiple health domains. The cognitive and physical functional health definitions revealed sweet spots for measures essential to maintain cognitive and physical functioning.

The model adjusted for the effect of lifestyle and socioeconomic factors identified sweet spot estimates for 60% of phenotypes (e.g., alanine aminotransferase and mean corpuscular volume), suggesting a substantial effect of physical activity level or nutrition risk on these phenotypes. The sweet spots for ferritin and free thyroxine were resistant to the adjustment with breakpoints highly correlated to the non-adjusted model, implying that these phenotypes may be less amenable to lifestyle modification. These initial observations indicate that the impact of lifestyle factors warrants further analysis with more advanced models in future work.

Although previous studies reported an association between low HbA1c (< 4%) and increased all-cause mortality [57], we did not initially detect optimal values for this phenotype for CLSA COM adults, yet post hoc analysis revealed a sweet spot estimate for nondiabetic males in white and Asian groups. This accords with earlier observations, which showed a J-shaped relationship between HbA1c and all-cause mortality in type-2 diabetic patients [58]. Our findings suggest that older Canadian adults on average have levels of HbA1c that are higher than a healthy level. Including younger adults would likely allow the regression model to detect sweet spots. In addition, our findings imply that controlling HbA1c is crucial for many aspects of health, including cognitive function.

Our results suggested a positive linear association between health decline and body measures. While it has been suggested that both overweight and underweight were linked to increased all-cause mortality in white adults [50], our model did not reveal an optimal value for hip or waist circumference for CLSA COM adults or for BMI for older females, potentially implying that too few participants were in the healthy range to detect a sweet spot. Our results for body measures serve as additional evidence of overweight and obesity as a health problem among older Canadian adults, and imply the importance of controlling body composition to maintain health.

In contrast to a widely held view that low LDL correlates with better health, we found that health deficit declined with increasing LDL for older adults, with the exception of an optimal value estimated using cognitive deficit for older females. However, this observed negative association is consistent with some previous population-based studies [59, 60].

Our results for hematology measures were consistent with observations in the Super-Seniors Study of heteroskedasticity for hemoglobin, mean corpuscular volume, and red blood cell distribution width, and the present study identified three additional hematology phenotypes with variance effect on health (white blood cells, granulocytes, and monocytes). Further confirmatory studies are needed to replicate sweet spots identified in CLSA measures.

### Limitations and strengths

Focusing our primary analyses on the largest ethnicity group (white), although it provides the greatest statistical power, limits our ability to generalize results across the entire Canadian population and other populations. With the exception of LDL, we were not able to replicate our findings in other ethnic groups for which sample sizes were substantially smaller. Due to CLSA exclusion criteria, such as living in long-term care institutions or cognitive impairment at the time of recruitment, our analysis of the baseline data was limited for detection of variance heterogeneity in cognitive functioning (IV). However, we expect to identify more traits with this instrument using CLSA follow-up data in the future. We also made assumptions that might be violated for some phenotypes. Specifically, we assumed the existence of a single optimal value for health-related phenotypes while the true association with health deficit might be more complex. We also made a number of assumptions to determine a subset of phenotypes for replication analysis in non-white groups.

The main strengths of our work are the novelty of the analysis and its potential clinical utility.

### Clinical implications

An important clinically relevant finding was that the 95% confidence intervals for the sweet spots of numerous phenotypes are much narrower than accepted clinical reference ranges. For example, for aspartate aminotransferase, we observed [8.4; 11.1] while the reference interval is reported as 8–41 U/L [61]. The difference might be explained by the approach used to develop reference intervals. Such intervals are commonly derived from case-control studies that compare the level of a phenotype between a healthy group and those with a specific disease. In contrast, we examined differences in health, which may detect more subtle dysregulation.

Our approach has the potential to inform and encourage interventions at early stages of dysregulation, before development of overt disease. For instance, viewing eGFR as a biomarker with a sweet spot allows a more nuanced view than the clinical view in which all values within the reference range are considered normal, and could serve to identify individuals who are not yet outside a clinically normal range but would benefit from earlier and individualized intervention (for example weight loss or reduction of sodium and sugar intake), to preserve kidney function and health.

One of the possible uses of the findings is constructing a per-participant representation of homeostatic dysregulation by summing the absolute difference of each phenotype from its corresponding sweet spot. In contrast to the deficit accumulation frailty index or allostatic load, such a metric would provide an unbiased score that reflects the overall health state and does not depend on diagnosis of conditions. Such a score would also be more accurate than physiological dysregulation, which is calculated as cumulative deviation from the means [17], because means do not necessarily coincide with optimal values, especially in a cohort of older adults.

Finally, a nuanced health report card for individuals based on deviation from optimal values could predict future health outcomes and be used to motivate individuals to take action before they are outside of the clinically normal ranges.

## Conclusion

Our study validates the proposed approach of using lower variance in healthier people as an indicator of health-relevant phenotypes. It also underscores the relevance of applying a homeostasis lens to human physiology. This approach, which is agnostic to prior understanding of the biological basis of a phenotype’s relevance to health, will be useful for analysis of phenotypes, such as diverse metabolomics measures that are not yet well-characterized or understood.

### Supplementary Information

Below is the link to the electronic supplementary material.

Supplementary file1 (DOCX 826 KB)

## Data Availability

Data are available from the Canadian Longitudinal Study on Aging (http://www.clsa-elcv.ca) for researchers who meet the criteria for access to de-identified CLSA data.

## Declarations

### Disclaimer

The opinions expressed in this manuscript are the authors’ own and do not reflect the views of the Canadian Longitudinal Study on Aging.
