# Supplementary material for: Physiological phenotypes have optimal values relevant to healthy aging: sweet spots deduced from the Canadian Longitudinal Study on Aging
Source: GeroScience. 2023 Sep 9;46(2):1589–605. doi: 10.1007/s11357-023-00895-2 (PMC10828371; doi:10.1007/s11357-023-00895-2)
Supplement: Supplementary file 1 — Supplementary file1 (DOCX 826 KB) [file 11357_2023_895_MOESM1_ESM.docx]

**Physiological phenotypes have optimal values relevant to healthy aging: Sweet spots deduced from the Canadian Longitudinal Study on Aging**

**SUPPLEMENTARY DATA**

**Figure S1.** **Health instruments.** Distribution of health deficit scores among self-reported white CLSA participants stratified by age groups (left), mean deficit score vs. age (right) for Instruments (A) I - the Frailty Index; (B) II - the number of five diseases: cancer (except non-melanoma skin cancer), cardiovascular disease, major pulmonary disease, dementia, and diabetes; (C) III - the number of other chronic conditions; (D) IV- composite cognitive score; (E) V- physical functioning. A higher score corresponds to worse health.

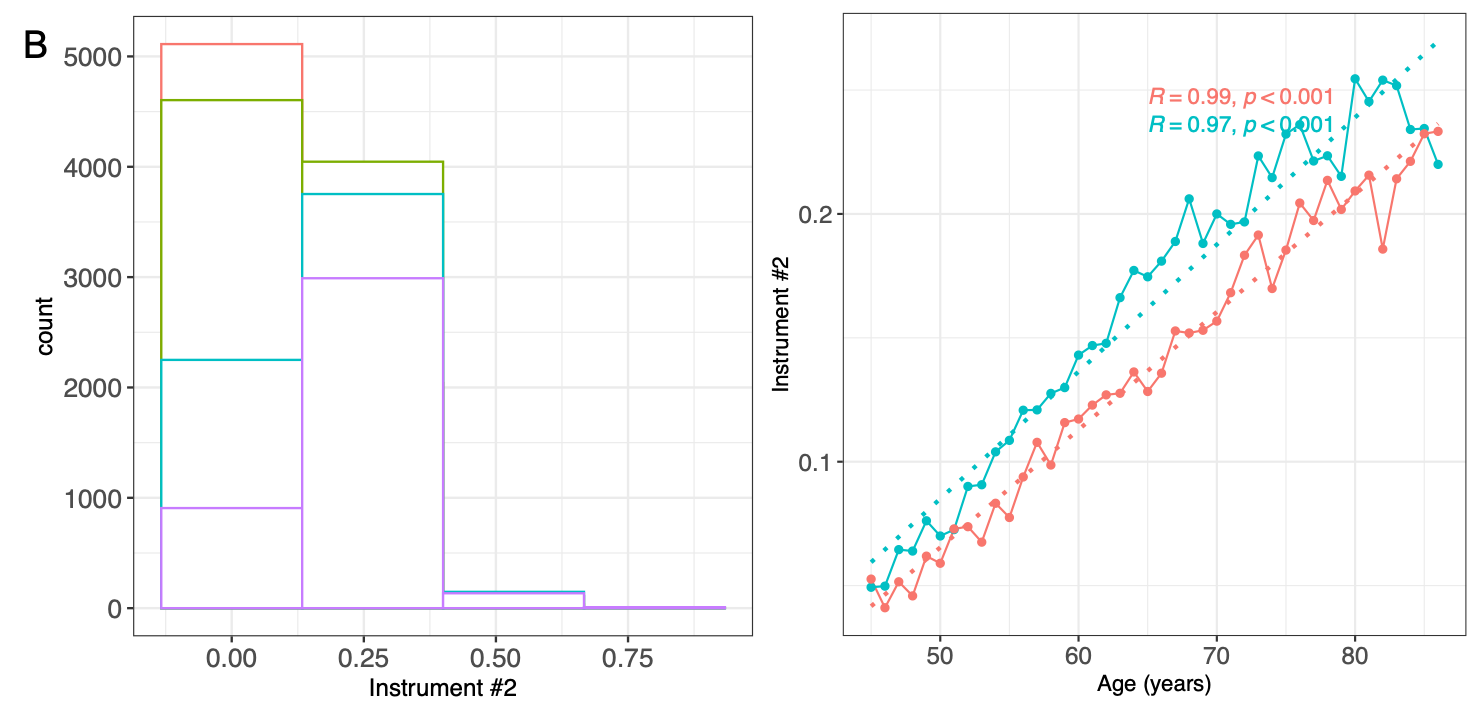

**Figure S2.** Venn diagram representing health-related phenotypes detected by a Brown-Forsythe test for heteroskedasticity. Results are from primary analysis on self-reported white CLSA participants. (A) for specific health Instrument: I - the Frailty Index; II - the number of five diseases: cancer (except non-melanoma skin cancer), cardiovascular disease, major pulmonary disease, dementia, and diabetes; III - the number of other chronic conditions; IV- composite cognitive score; V- physical functioning. A phenotype detected by all Instruments is Hemoglobin A1c, %. (B) for specific sex

**A** **B**


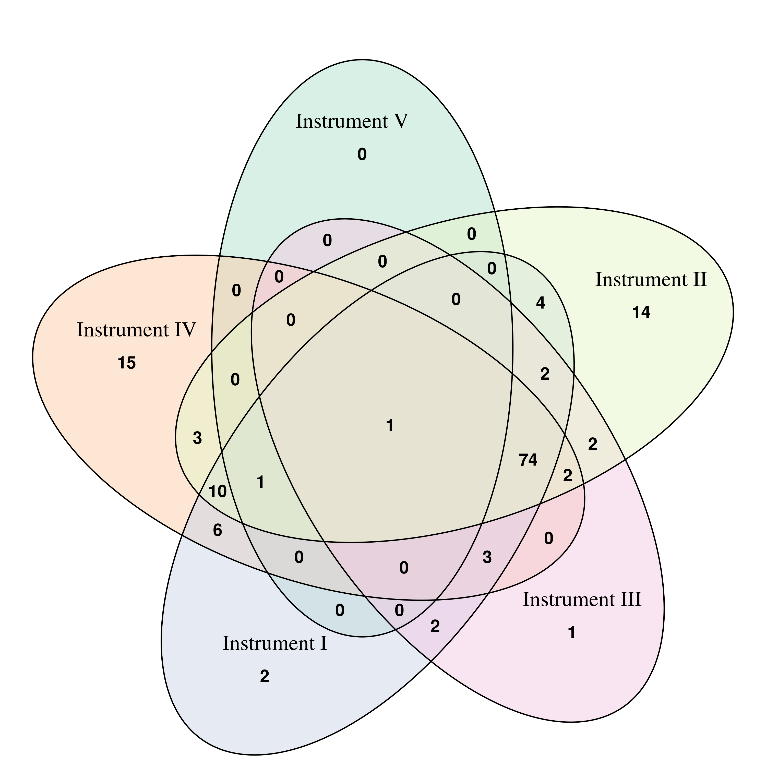

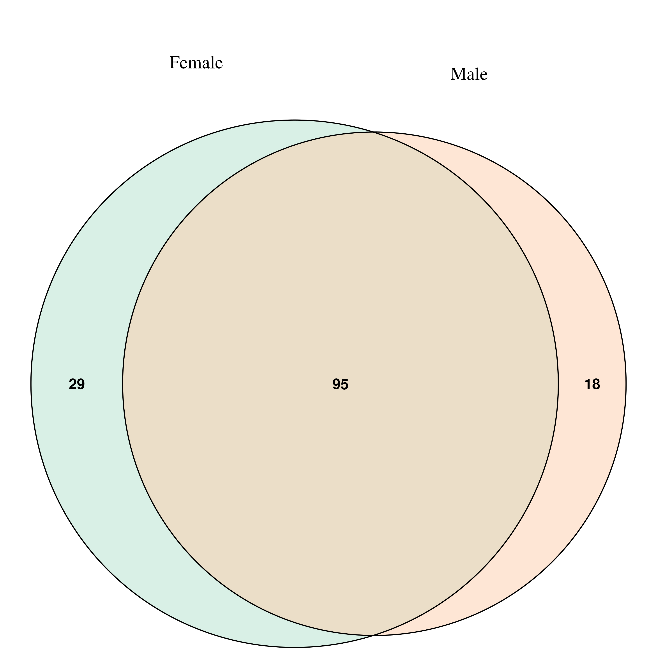


**Figure S3.** Significant segmented regression results obtained for the major ethnic group (white). (A) Histogram of estimated sweet spot position across phenotypes, sexes and instruments. The range was -1.58 to 2.51 (mean -0.24) (B) Histogram of differences in slopes across phenotypes, sexes and instruments. The range was from 0.008 to 0.605 (mean 0.034).

**A** **B**

**Figure S4.** Power calculation for replication analysis performed using the R package *segmented* (A) Asian ethnic group. Parameters used to calculate power: sample size=282; the response standard deviation 0.07; the slope difference across phenotypes that showed optimal values ranged from 0.008 to 0.605; the breakpoint position -0.24. Estimated slope difference cut off was 0.042. (B) Black ethnic group. Parameters used to calculate power: sample size=95; the response standard deviation 0.07; the slope difference across phenotypes that showed optimal values ranged from 0.008 to 0.605; the breakpoint position -0.24. Estimated slope difference cut-off is 0.068.

**A** **B**

**Table S1**. CLSA phenotypes selected for Sweet Spot testing

| **Category** | **Phenotype** |
| --- | --- |
|  |  |
| Body-composition analysis |  |
|  | Grams of Fat tissue in Head region |
|  | Grams of Lean tissue in Head region |
|  | Mass in grams in Head region |
|  | Percentage of fat tissue to total tissue mass in Head region |
|  | Grams of Fat tissue in Left arm region |
|  | Grams of Lean tissue in Left arm region |
|  | Mass in grams in Left arm region |
|  | Percentage of fat tissue to total tissue mass in Left arm region |
|  | Grams of Fat tissue in Right arm region |
|  | Grams of Lean tissue in Right arm region |
|  | Mass in grams in Right arm region |
|  | Percentage of fat tissue to total tissue mass in Right arm region |
|  | Grams of Fat tissue in Trunk region |
|  | Grams of Lean tissue in Trunk region |
|  | Mass in grams in Trunk region |
|  | Percentage of fat tissue to total tissue mass in Trunk region |
|  | Grams of Fat tissue in Left leg region |
|  | Grams of Lean tissue in Left leg region |
|  | Mass in grams in Left leg region |
|  | Percentage of fat tissue to total tissue mass in Left leg region |
|  | Grams of Fat tissue in Right leg region |
|  | Grams of Lean tissue in Right leg region |
|  | Mass in grams in Right leg region |
|  | Percentage of fat tissue to total tissue mass in Right leg region |
|  | Grams of Fat tissue in Summary |
|  | Grams of Lean tissue in Summary |
|  | Mass in grams in Summary |
|  | Percentage of fat tissue to total tissue mass in Summary |
|  | Grams of Fat tissue in all included regions |
|  | Grams of Lean tissue in all included regions |
|  | Mass in grams in all included regions |
|  | Percentage of fat tissue to total tissue mass in all included regions |
|  | % of total body mass that is fat |
|  | Total body mass divided by height squared (kg/m2) |
|  | % fat in Android region / % fat in Gynoid region |
|  | % fat in Android region |
|  | % fat in Gynoid region |
|  | % fat in Trunk / % fat in legs |
|  | Trunk fat / Limb fat |
|  | Total fat mass divided by height squared (kg/m2) |
|  | Total fat mass in whole body |
|  | Total lean mass divided by height squared (kg/m2) |
|  | Lean mass in limbs divided by height squared (kg/m2) |
|  | Total lean mass in whole body |
|  | Total lean mass without bone, divided by height squared (kg/m2) |
|  | Lean mass without bone in limbs, divided by height squared (kg/m2) |
|  | Total lean mass without bone in whole body (in grams) |
|  | Fat mass in grams (Android + Gynoid regions) |
|  | Lean mass in grams (Android + Gynoid regions). |
|  | Fat + Lean mass in grams (Android + Gynoid regions) |
|  | Percent Fat (Android + Gynoid regions) |
|  | Fat Tissue in Android region (grams) |
|  | Lean Tissue in Android region (grams) |
|  | All Tissue in Android region (grams) |
|  | Percent fat in Android region |
|  | Fat Tissue in Gynoid region (grams) |
|  | Lean Tissue in Gynoid region (grams) |
|  | All Tissue in Gynoid region (grams) |
|  | Percent fat in Gynoid region |
|  | Fat Tissue in largest Visceral Fat region (grams) |
|  | Lean Tissue in largest Visceral Fat region (grams) |
|  | All Tissue in largest Visceral Fat region (grams) |
|  | Percent fat in largest Visceral Fat region |
|  | Fat Tissue in middle Visceral Fat region (grams) |
|  | Lean Tissue in middle Visceral Fat region (grams) |
|  | All Tissue in middle Visceral Fat region (grams) |
|  | Percent fat in middle Visceral Fat region |
|  | Fat Tissue in inner-most Visceral Fat region (grams) |
|  | Lean Tissue in inner-most Visceral Fat region (grams) |
|  | All Tissue in inner-most Visceral Fat region (grams) |
|  | Percent fat in inner-most Visceral Fat region |
|  | Cross-sectional area (cm squared) of fat inside abdominal cavity |
|  | Mass of fat (grams) inside abdominal cavity (in test region) |
|  | Volume of fat (cc) inside abdominal cavity (in test region) |
|  | Cross-sectional area (cm squared) of all fat in test region |
|  | Cross-sectional area (cm squared) of fat outside abdominal cavit |
|  | Width of largest Visceral Fat region (patient width) |
|  | Width of middle Visceral Fat region (width of outside of abdominal cavity |
|  | Width of inner-most Visceral Fat region (width of inside of abdominal cavity) |
|  | Pure lean mass (without bone) in left arm |
|  | Pure lean mass (without bone) in right arm |
|  | Pure lean mass (without bone) in left leg |
|  | Pure lean mass (without bone) in right leg |
|  | Pure lean mass (without bone) in head |
|  | Pure lean mass (without bone) in trunk |
|  | Total pure lean mass (without bone) excluding head region |
|  | Pure lean mass (without bone) in limbs |
| Body measures |  |
|  | Average weight in kg |
|  | Body Mass Index |
|  | Average height in m |
|  | Waist circumference in cm |
|  | Hips circumference in cm |
|  | Waist to hip ratio |
| Bone mineral density: hip region |  |
|  | Area for Trochanter Bone analysis - Right hip |
|  | BMC for Trochanter Bone analysis - Right hip |
|  | BMD for Trochanter Bone analysis - Right hip |
|  | Area for Trochanter Bone analysis - Left hip |
|  | BMC for Trochanter Bone analysis - Left hip |
|  | BMD for Trochanter Bone analysis - Left hip |
|  | Area for inter -Trochanter Bone analysis - Right hip |
|  | BMD for inter -Trochanter Bone analysis - Right hip |
|  | Area for inter -Trochanter Bone analysis - left hip |
|  | BMD for inter -Trochanter Bone analysis - left hip |
|  | BMC for inter -Trochanter Bone analysis - right hip |
|  | BMC for inter -Trochanter Bone analysis - left hip |
|  | Area for Neck Bone analysis - right hip |
|  | BMC for Neck Bone analysis - right hip |
|  | BMD for Neck Bone analysis - right hip |
|  | Area for Neck Bone analysis - left hip |
|  | BMC for Neck Bone analysis - left hip |
|  | BMD for Neck Bone analysis - left hip |
|  | Area for Ward’s Triangle Bone analysis - right hip |
|  | BMC for Ward’s Triangle Bone analysis - right hip |
|  | BMD for Ward’s Triangle Bone analysis - right hip |
|  | Area for Ward’s Triangle Bone analysis - left hip |
|  | BMC for Ward’s Triangle Bone analysis - left hip |
|  | BMD for Ward’s Triangle Bone analysis - left hip |
|  | Area for Total Bone analysis for all included regions - right hip |
|  | BMC for Total Bone analysis for all included regions - right hip |
|  | BMD for Total Bone analysis for all included regions - right hip |
|  | Area for Total Bone analysis for all included regions - left hip |
|  | BMC for Total Bone analysis for all included regions - left hip |
|  | BMD for Total Bone analysis for all included regions - left hip |
|  | Width of ROI in cm - Right hip |
|  | Width of ROI in cm - left hip |
|  | Height of ROI in cm - Right hip |
|  | Height of ROI in cm - Left hip |
|  | Length of Right Hip Axis in cm |
|  | Length of Left Hip Axis in cm |
|  | Narrow Neck Bone Mineral Density (g/cm2) -Right hip |
|  | Narrow Neck Bone Mineral Density (g/cm2) -Left hip |
|  | Narrow Neck Cross Sectional Area (cm2) - left hip |
|  | Narrow Neck Cross Sectional Moment of Inertia (cm4) - left hip |
|  | Narrow Neck Subperiosteal Width (cm) - left hip |
|  | Narrow Neck Endocortical Diameter (cm) - left hip |
|  | Narrow Neck Average Cortical Thickness (cm) - left hip |
|  | Narrow Neck Profile Center Distance (cm) - left hip |
|  | Narrow Neck Center of Mass Position (dimensionless) - left hip |
|  | Narrow Neck Section Modulus (cm3) - left hip |
|  | Narrow Neck Buckling Ratio (dimensionless) - left hip |
|  | Narrow Neck Cross Sectional Area (cm2) -right hip |
|  | Narrow Neck Cross Sectional Moment of Inertia (cm4) -right hip |
|  | Narrow Neck Subperiosteal Width (cm) -right hip |
|  | Narrow Neck Endocortical Diameter (cm) -right hip |
|  | Narrow Neck Average Cortical Thickness (cm) -right hip |
|  | Narrow Neck Profile Center Distance (cm) -right hip |
|  | Narrow Neck Center of Mass Position (dimensionless) -right hip |
|  | Narrow Neck Section Modulus (cm3) -right hip |
|  | Narrow Neck Buckling Ratio (dimensionless) -right hip |
|  | Intertrochanter Cross Sectional Area (cm2) - left hip |
|  | Intertrochanter Cross Sectional Moment of Inertia (cm4) - left hip |
|  | Intertrochanter Subperiosteal Width (cm) - left hip |
|  | Intertrochanter Endocortical Diameter (cm) - left hip |
|  | Intertrochanter Average Cortical Thickness (cm) - left hip |
|  | Intertrochanter Profile Center Distance (cm) - left hip |
|  | Intertrochanter Center of Mass Position (dimensionless) - left hip |
|  | Intertrochanter Section Modulus (cm3) - left hip |
|  | Intertrochanter Buckling Ratio (dimensionless) - left hip |
|  | Intertrochanter Cross Sectional Area (cm2) -right hip |
|  | Intertrochanter Cross Sectional Moment of Inertia (cm4) -right hip |
|  | Intertrochanter Subperiosteal Width (cm) -right hip |
|  | Intertrochanter Endocortical Diameter (cm) -right hip |
|  | Intertrochanter Average Cortical Thickness (cm) -right hip |
|  | Intertrochanter Profile Center Distance (cm) -right hip |
|  | Intertrochanter Center of Mass Position (dimensionless) -right hip |
|  | Intertrochanter Section Modulus (cm3) -right hip |
|  | Intertrochanter Buckling Ratio (dimensionless) -right hip |
|  | Intertrochanter Bone Mineral Density (g/cm2) - right hip |
|  | Intertrochanter Bone Mineral Density (g/cm2) - left hip |
|  | Femur Shaft Cross Sectional Area (cm2) - left hip |
|  | Femur Shaft Cross Sectional Moment of Inertia (cm4) - left hip |
|  | Femur Shaft Subperiosteal Width (cm) - left hip |
|  | Femur Shaft Endocortical Diameter (cm) - left hip |
|  | Femur Shaft Average Cortical Thickness (cm) - left hip |
|  | Femur Shaft Profile Center Distance (cm) - left hip |
|  | Femur Shaft Center of Mass Position (dimensionless) - left hip |
|  | Femur Shaft Section Modulus (cm3) - left hip |
|  | Femur Shaft Buckling Ratio (dimensionless) - left hip |
|  | Femur Shaft Cross Sectional Area (cm2) -right hip |
|  | Femur Shaft Cross Sectional Moment of Inertia (cm4) -right hip |
|  | Femur Shaft Subperiosteal Width (cm) -right hip |
|  | Femur Shaft Endocortical Diameter (cm) -right hip |
|  | Femur Shaft Average Cortical Thickness (cm) -right hip |
|  | Femur Shaft Profile Center Distance (cm) -right hip |
|  | Femur Shaft Center of Mass Position (dimensionless) -right hip |
|  | Femur Shaft Section Modulus (cm3) -right hip |
|  | Femur Shaft Buckling Ratio (dimensionless) -right hip |
|  | Femur Shaft Bone Mineral Density (g/cm2) - right hip |
|  | Femur Shaft Bone Mineral Density (g/cm2) - left hip |
|  | Angle formed by shaft center line and neck axis in degrees - right hip |
|  | Angle formed by shaft center line and neck axis in degrees - left hip |
| Bone mineral density: whole body |  |
|  | Total body area |
|  | Total BMC |
|  | Total BMD |
|  | Total bone area excluding the head region |
|  | Total BMC excluding the head region |
|  | Total BMD excluding the head region |
|  | Bone area for the Head region Bone analysis |
|  | Bone mineral content for the Head region |
|  | Bone mineral density for the Head region |
|  | Bone area for the Left Arm region |
|  | Bone mineral content for the Left Arm region |
|  | Bone mineral density for the Left Arm region |
|  | Bone area for the Right Arm region |
|  | Bone mineral content for the Right Arm region |
|  | Bone mineral density for the Right Arm region |
|  | Bone area for the Left Rib region |
|  | Bone mineral content for the Left Rib region |
|  | Bone mineral density for the Left Rib region |
|  | Bone area for the Right Rib region |
|  | Bone mineral content for the Right Rib region |
|  | Bone mineral density for the Right Rib region |
|  | Bone area for the Thoracic region |
|  | Bone mineral content for the Thoracic region |
|  | Bone mineral density for the Thoracic region |
|  | Bone area for the Lumbar Spine region |
|  | Bone mineral content for the Lumbar Spine region |
|  | Bone mineral density for the Lumbar Spine region |
|  | Bone area for the Pelvic region |
|  | Bone mineral content for the Pelvic region |
|  | Bone mineral density for the Pelvic region |
|  | Bone area for the Left Leg region |
|  | Bone mineral content for the Left Leg region |
|  | Bone mineral density for the Left Leg region |
|  | Bone area for the Right Leg region |
|  | Bone mineral content for the Right Leg region |
|  | Bone mineral density for the Right Leg region |
| Blood chemistry |  |
|  | Hemoglobin A1c, % |
|  | 25-hydroxyvitamin D, nmol/L |
|  | Albumin, g/L |
|  | Alanine Aminotransferase, U/L |
|  | Creatinine, µmol/L |
|  | Ferritin, µg/L |
|  | Free Thyroxine, pmol/L |
|  | Cholesterol, mmol/L |
|  | High-Density Lipoprotein, mmol/L |
|  | Low-Density Lipoprotein, calculated, mmol/L |
|  | non HDL, mmol/L |
|  | Triglycerides, mmol/L |
|  | Thyroid-Stimulating Hormone, mIU/L |
|  | Estimated Glomerular Filtration Rate, mL/min/1.73m^2 |
| Electrocardiogram |  |
|  | Ventricular Rate, BPM |
|  | PQ Interval, ms |
|  | P Duration, ms |
|  | QRS Duration, ms |
|  | QT Interval, ms |
|  | QTC Interval, ms |
|  | RR Interval, ms |
|  | PP Interval, ms |
|  | P Axis, degree |
|  | R Axis, degree |
|  | T Axis, degree |
|  | QRS Number |
|  | P Onset, ms |
|  | P Offset, ms |
|  | Q Onset, ms |
|  | Q Offset, ms |
|  | T Offset, ms |
| Hematology |  |
|  | White blood cells, 10^9^/L |
|  | Lymphocytes (relative number), % |
|  | Monocytes (relative number), % |
|  | Granulocytes (relative number), % |
|  | Lymphocytes (absolute number), 10^9^/L |
|  | Monocytes (absolute number), 10^9^/L |
|  | Granulocytes (absolute number), 10^9^/L |
|  | Red blood cells, 10^12^/L |
|  | Hemoglobin, g/L |
|  | Hematocrit, L/L |
|  | Mean corpuscular volume, fL |
|  | Mean corpuscular hemoglobin, pg |
|  | Mean corpuscular hemoglobin concentration, g/L |
|  | Red blood cell distribution width, % |
|  | Platelets, 10^9^/L |
|  | Mean platelet volume, fL |
| Inflammatory biomarkers |  |
|  | High Sensitivity C-Reactive Protein, mg/L |
|  | Tumor Necrosis Factor - Alpha (TNF-a), pg/mL |
|  | Interleukin-6 (IL-6), pg/mL |
| Vital signs |  |
|  | Average systolic blood pressure (excluding 1st reading) |
|  | Average diastolic blood pressure (excluding 1st reading) |
|  | Average pluse rate (excluding 1st reading) |

**Table S2.** Self-reported phenotypes and assessment tests used to construct health Instruments and methods of operationalization.

| **Instrument*** | **Phenotype** | **Methods of operationalization** |
| --- | --- | --- |
| **I** |  |  |
|  | Self-rated health | Poor=1; Fair=0.75; Good 0.5; Very good 0.25; Excellent=0 |
|  | Self-rated vision |  |
|  | Self-rated hearing |  |
|  | steoarthritis in the hip, in the knee, in one or both hands | osteoart = 0 if no for all types; osteoart = 1 if yes for any type |
|  | Other type of arthritis, Rheumatoid arthritis | arthritis = 0 if no for all types; arthritis = 1 if yes for any type |
|  | Emphysema, chronic bronchitis, COPD, or chronic changes in lungs due to smoking | yes = 1; no = 0 |
|  | Diabetes, borderline diabetes or blood sugar is high |  |
|  | Heart disease (including congestive heart failure, or CHF) |  |
|  | Angina |  |
|  | Heart attack or myocardial infarction |  |
|  | Peripheral vascular disease or poor circulation in limbs |  |
|  | Stroke or CVA |  |
|  | Experienced a ministroke or TIA |  |
|  | Memory problem |  |
|  | Parkinsonism or Parkinsons Disease |  |
|  | Intestinal or stomach ulcers |  |
|  | Bowel disorder |  |
|  | Bowel incontinence |  |
|  | Urinary incontinence |  |
|  | Ever had cataracts |  |
|  | Ever had glaucoma |  |
|  | Macular degeneration |  |
|  | Cancer |  |
|  | Osteoporosis |  |
|  | Back problems excluding fibromyalgia and arthritis |  |
|  | UNDER-active thyroid gland |  |
|  | OVER-active thyroid gland (hyperthyroidism) |  |
|  | Kidney disease or kidney failure |  |
|  | Pneumonia - past year |  |
|  | Urinary Tract Infection - past year |  |
|  | dementia or Alzheimers disease |  |
|  | Falls in the last 12 months; Number of falls in last 12 months | none = 0; only one = 0.5; two or more = 1 |
|  | OARS scale: Able to walk; OARS scale: Able to walk with help; OARS scale: Unable to walk | unable=1; able with help = 0.5; able = 0 |
|  | OARS scale: Able to take bath; OARS scale: Able to take bath with help; OARS scale: Unable to take bath |  |
|  | OARS scale: Able to go shopping; OARS scale: Able to go shopping with help; OARS scale: Unable to go shopping |  |
|  | OARS scale: Able to do housework; OARS scale: Able to do housework with help; OARS scale: Unable to do housework |  |
|  | OARS scale: Able to dress; OARS scale: Able to dress with help; OARS scale: Unable to dress |  |
|  | OARS scale: Able to take care of appearance; OARS scale: Able to take care of appearance with help; OARS scale: Unable to take care of appearance |  |
|  | OARS scale: Able to get out of bed; OARS scale: Able to get out of bed with help; OARS scale: Unable to get out of bed |  |
|  | OARS scale: Able to use telephone; OARS scale: Able to use telephone with help; OARS scale: Unable to use telephone |  |
|  | OARS scale: Able to travel; OARS scale: Able to travel with help; OARS scale: Unable to travel |  |
|  | OARS scale: Able to prepare meals; OARS scale: Able to prepare meals with help; OARS scale: Unable to prepare meals |  |
|  | OARS scale: Able to take medicine; OARS scale: Able to take medicine with help; OARS scale: Unable to take medicine |  |
|  | OARS scale: Able to handle money; OARS scale: Able to handle money with help; OARS scale: Unable to handle money |  |
|  | Verbal fluency | 0 if RNS <0.85; 1 if RNS >0.85 |
|  | Executive function |  |
|  | Immediate recall |  |
|  | Delayed recall |  |
|  | CES-D 10 scale: Frequency feel everything is an effort | All of the time =1, Occasionally=0.66, Some of the time=0.33, Rarely or never=1 |
|  | CES-D 10 scale: Frequency feel lonely |  |
|  | CES-D 10 scale: Frequency feel could not 'get going' |  |
| **II** |  |  |
|  | Cancer; Skin cancer: non-melanoma; skin cancer: not specified; | individuals with unknown skin cancer without other diagnosis marked as NA. cancer.score=0 if Cancer=yes and non-melanoma = no. cancer.score=1 for other types. |
|  | Dementia or Alzheimer's disease | yes = 1; no = 0 |
|  | Emphysema, chronic bronchitis, COPD, or chronic changes in lungs due to smoking |  |
|  | Diabetes, borderline diabetes or blood sugar is high |  |
|  | Heart attack or myocardial infarction; angina; Stroke or CVA; Heart disease (including congestive heart failure, or CHF); Peripheral vascular disease or poor circulation in limbs; Experienced a ministroke or TIA; Thoracic, abdominal or cerebral aneurysm; Blockage in your arteries; Coronary artery bypass surgery; Undergoing other treatment for stroke; Undergoing other treatment for ministroke; Currently taking anticoagulants; Currently taking drugs for ischemic heart disease; Currently taking drugs for stroke | 0 if no for all types; 1 if yes for any type |
| **III** |  |  |
|  | Anxiety disorder | yes = 1; no = 0 |
|  | Other type of arthritis |  |
|  | Asthma |  |
|  | Back problems excluding fibromyalgia and arthritis |  |
|  | Other infections - past year |  |
|  | Epilepsy |  |
|  | Kidney disease or kidney failure |  |
|  | Mood disorder |  |
|  | Multiple sclerosis |  |
|  | Osteoarthritis in one or both hands |  |
|  | Osteoarthritis in the hip |  |
|  | Osteoarthritis in the knee |  |
|  | Osteoporosis |  |
|  | OVER-active thyroid gland (hyperthyroidism) |  |
|  | Parkinsonism or Parkinson's Disease |  |
|  | Rheumatoid arthritis |  |
|  | Intestinal or stomach ulcers |  |
|  | UNDER-active thyroid gland |  |
|  | Clinical depression |  |
|  | Ever had cataracts |  |
|  | Ever had glaucoma |  |
|  | Positive Screen for Traumatic Brain Injury (TBI) |  |
|  | Macular degeneration |  |
|  | Migraine headaches |  |
|  | Ear infections | 0 if no for all types; 1 if yes for any type |
|  | Eye infections |  |
|  | Bowel disorder; Bowel incontinence |  |
|  | Urinary incontinence; Urinary Tract Infection - past year |  |
| **IV** |  | RNS |
|  | REYI - Number of words (or variants) correctly recalled in 90 seconds - Immediate Recall | Direction of risk: inverse |
|  | AFT - Score 1 - Number of different animals recited in 60 seconds | Direction of risk: inverse |
|  | MAT - Number of correct consecutive numeric and alphabetical alternations in 30 seconds | Direction of risk: inverse |
|  | PMT (Event-based Prospective Memory Test) | Direction of risk: inverse |
|  | TMT (Prospective Memory Test) | Direction of risk: inverse |
|  | Stroop Neurological Screening Test. Colored dots | Direction of risk: direct |
|  | Stroop Neurological Screening Test. Common words printed in same colors as dots | Direction of risk: direct |
|  | Stroop Neurological Screening Test. Color words printed in non-corresponding colors of ink | Direction of risk: direct |
|  | FAS (Controlled Oral Word Association Test). “F” words | Direction of risk: inverse |
|  | FAS (Controlled Oral Word Association Test). “A” words | Direction of risk: inverse |
|  | FAS (Controlled Oral Word Association Test). “S” words | Direction of risk: inverse |
|  | CRT (Choice Reaction Time Test) | Direction of risk: direct |
| **V** |  | RNS |
|  | Best attained time - Standing Balance | Direction of risk: inverse *** |
|  | Average time for 1 chair rise (in seconds) | Direction of risk: direct |
|  | Average grip strength for all trials | Direction of risk: inverse |
|  | Total time required to complete Timed Get Up and Go (in seconds) | Direction of risk: direct |
|  | Total time required to complete 4mWalk (in seconds) | Direction of risk: direct |

* Instruments: I - the Frailty Index; II - the number of five diseases: cancer (except non-melanoma skin cancer), cardiovascular disease, major pulmonary disease, dementia, and diabetes; III - the number of other chronic conditions; IV - composite cognitive score; V- physical functioning

** RNS – The rank normalized score

*** The inverse direction of risk means that a higher assessment test score corresponds to better performance

**Table S3.** Missingness rate (MR) and sample size (SZ) for each phenotype analyzed

| **Phenotype** | **White group** | | | | | **Asian group** | | | | **Black group** | | | | |
| --- | --- | --- | --- | --- | --- | --- | --- | --- | --- | --- | --- | --- | --- | --- |
|  | **Females** | | | **Males** | | **Females** | | **Males** | | **Females** | | **Males** | | |
|  | SZ | MR | SZ | | MR | SZ | MR | SZ | MR | SZ | MR | | SZ | MR |
| Albumin | 12881 | 0.103 | 12664 | | 0.103 | 215 | 0.103 | 317 | 0.103 | 89 | 0.103 | | 89 | 0.103 |
| Alanine Aminotransferase | 12879 | 0.103 | 12661 | | 0.103 | 215 | 0.103 | 317 | 0.103 | 89 | 0.103 | | 89 | 0.103 |
| Cholesterol | 12881 | 0.103 | 12664 | | 0.103 | 215 | 0.103 | 317 | 0.103 | 89 | 0.103 | | 89 | 0.103 |
| Creatinine | 12881 | 0.103 | 12664 | | 0.103 | 215 | 0.103 | 317 | 0.103 | 89 | 0.103 | | 89 | 0.103 |
| Estimated Glomerular Filtration Rate | 12881 | 0.103 | 12664 | | 0.103 | 215 | 0.103 | 317 | 0.103 | 89 | 0.103 | | 89 | 0.103 |
| Ferritin | 12872 | 0.103 | 12661 | | 0.103 | 215 | 0.103 | 317 | 0.103 | 89 | 0.103 | | 89 | 0.103 |
| Free Thyroxine | 12879 | 0.103 | 12663 | | 0.103 | 215 | 0.103 | 317 | 0.103 | 89 | 0.103 | | 89 | 0.103 |
| Granulocytes (absolute number) | 12113 | 0.155 | 11913 | | 0.155 | 207 | 0.155 | 309 | 0.155 | 82 | 0.155 | | 78 | 0.155 |
| Granulocytes (relative number) | 12113 | 0.155 | 11913 | | 0.155 | 207 | 0.155 | 309 | 0.155 | 82 | 0.155 | | 78 | 0.155 |
| Hemoglobin A1c | 12763 | 0.106 | 12686 | | 0.106 | 215 | 0.106 | 314 | 0.106 | 87 | 0.106 | | 87 | 0.106 |
| Hematocrit | 12113 | 0.155 | 11913 | | 0.155 | 207 | 0.155 | 309 | 0.155 | 82 | 0.155 | | 78 | 0.155 |
| High-Density Lipoprotein | 12881 | 0.103 | 12664 | | 0.103 | 215 | 0.103 | 317 | 0.103 | 89 | 0.103 | | 89 | 0.103 |
| Hemoglobin | 12113 | 0.155 | 11913 | | 0.155 | 207 | 0.155 | 309 | 0.155 | 82 | 0.155 | | 78 | 0.155 |
| High Sensitivity C-Reactive Protein | 12881 | 0.103 | 12663 | | 0.103 | 215 | 0.103 | 317 | 0.103 | 89 | 0.103 | | 89 | 0.103 |
| Interleukin-6 (IL-6) | 4706 | 0.677 | 4467 | | 0.677 | 88 | 0.677 | 119 | 0.677 | 28 | 0.677 | | 26 | 0.677 |
| Low-Density Lipoprotein, calculated | 12725 | 0.122 | 12277 | | 0.122 | 205 | 0.122 | 305 | 0.122 | 89 | 0.122 | | 89 | 0.122 |
| Lymphocytes (absolute number) | 12113 | 0.155 | 11913 | | 0.155 | 207 | 0.155 | 309 | 0.155 | 82 | 0.155 | | 78 | 0.155 |
| Lymphocytes (relative number) | 12113 | 0.155 | 11913 | | 0.155 | 207 | 0.155 | 309 | 0.155 | 82 | 0.155 | | 78 | 0.155 |
| Mean corpuscular hemoglobin concentration | 12113 | 0.155 | 11913 | | 0.155 | 207 | 0.155 | 309 | 0.155 | 82 | 0.155 | | 78 | 0.155 |
| Mean corpuscular hemoglobin | 12113 | 0.155 | 11913 | | 0.155 | 207 | 0.155 | 309 | 0.155 | 82 | 0.155 | | 78 | 0.155 |
| Mean corpuscular volume | 12113 | 0.155 | 11913 | | 0.155 | 207 | 0.155 | 309 | 0.155 | 82 | 0.155 | | 78 | 0.155 |
| Monocytes (absolute number) | 12113 | 0.155 | 11913 | | 0.155 | 207 | 0.155 | 309 | 0.155 | 82 | 0.155 | | 78 | 0.155 |
| Monocytes (relative number) | 12113 | 0.155 | 11913 | | 0.155 | 207 | 0.155 | 309 | 0.155 | 82 | 0.155 | | 78 | 0.155 |
| Mean platelet volume | 12113 | 0.155 | 11913 | | 0.155 | 207 | 0.155 | 309 | 0.155 | 82 | 0.155 | | 78 | 0.155 |
| non HDL | 12881 | 0.103 | 12664 | | 0.103 | 215 | 0.103 | 317 | 0.103 | 89 | 0.103 | | 89 | 0.103 |
| Platelets | 12113 | 0.155 | 11913 | | 0.155 | 207 | 0.155 | 309 | 0.155 | 82 | 0.155 | | 78 | 0.155 |
| Red blood cells | 12113 | 0.155 | 11913 | | 0.155 | 207 | 0.155 | 309 | 0.155 | 82 | 0.155 | | 78 | 0.155 |
| Red blood cell distribution width | 12113 | 0.155 | 11913 | | 0.155 | 207 | 0.155 | 309 | 0.155 | 82 | 0.155 | | 78 | 0.155 |
| Tumor Necrosis Factor - Alpha (TNF-a) | 4595 | 0.683 | 4411 | | 0.683 | 87 | 0.683 | 117 | 0.683 | 28 | 0.683 | | 26 | 0.683 |
| Triglycerides | 12881 | 0.103 | 12664 | | 0.103 | 215 | 0.103 | 317 | 0.103 | 89 | 0.103 | | 89 | 0.103 |
| Thyroid-Stimulating Hormone | 12853 | 0.104 | 12655 | | 0.104 | 215 | 0.104 | 317 | 0.104 | 88 | 0.104 | | 88 | 0.104 |
| 25-hydroxyvitamin D | 12878 | 0.103 | 12660 | | 0.103 | 215 | 0.103 | 317 | 0.103 | 89 | 0.103 | | 89 | 0.103 |
| White blood cells | 12113 | 0.155 | 11913 | | 0.155 | 207 | 0.155 | 309 | 0.155 | 82 | 0.155 | | 78 | 0.155 |
| Average diastolic blood pressure (excluding 1st reading) | 14351 | 0.010 | 13754 | | 0.010 | 266 | 0.010 | 356 | 0.010 | 110 | 0.010 | | 110 | 0.010 |
| Average pluse rate (excluding 1st reading) | 14351 | 0.010 | 13751 | | 0.010 | 266 | 0.010 | 356 | 0.010 | 110 | 0.010 | | 110 | 0.010 |
| Average systolic blood pressure (excluding 1st reading) | 14351 | 0.010 | 13754 | | 0.010 | 266 | 0.010 | 356 | 0.010 | 110 | 0.010 | | 110 | 0.010 |
| Fat Tissue in Android region (grams) | 13886 | 0.043 | 13259 | | 0.043 | 260 | 0.043 | 349 | 0.043 | 105 | 0.043 | | 104 | 0.043 |
| Lean Tissue in Android region (grams) | 13886 | 0.043 | 13259 | | 0.043 | 260 | 0.043 | 349 | 0.043 | 105 | 0.043 | | 104 | 0.043 |
| All Tissue in Android region (grams) | 13886 | 0.043 | 13259 | | 0.043 | 260 | 0.043 | 349 | 0.043 | 105 | 0.043 | | 104 | 0.043 |
| Percent fat in Android region | 13886 | 0.043 | 13259 | | 0.043 | 260 | 0.043 | 349 | 0.043 | 105 | 0.043 | | 104 | 0.043 |
| Width of largest Visceral Fat region (patient width) | 13886 | 0.043 | 13259 | | 0.043 | 260 | 0.043 | 349 | 0.043 | 105 | 0.043 | | 104 | 0.043 |
| Width of inner-most Visceral Fat region (width of inside of abdominal cavity) | 13886 | 0.043 | 13259 | | 0.043 | 260 | 0.043 | 349 | 0.043 | 105 | 0.043 | | 104 | 0.043 |
| Fat Tissue in Gynoid region (grams) | 13886 | 0.043 | 13259 | | 0.043 | 260 | 0.043 | 349 | 0.043 | 105 | 0.043 | | 104 | 0.043 |
| Lean Tissue in Gynoid region (grams) | 13886 | 0.043 | 13259 | | 0.043 | 260 | 0.043 | 349 | 0.043 | 105 | 0.043 | | 104 | 0.043 |
| All Tissue in Gynoid region (grams) | 13886 | 0.043 | 13259 | | 0.043 | 260 | 0.043 | 349 | 0.043 | 105 | 0.043 | | 104 | 0.043 |
| Percent fat in Gynoid region | 13886 | 0.043 | 13259 | | 0.043 | 260 | 0.043 | 349 | 0.043 | 105 | 0.043 | | 104 | 0.043 |
| Width of middle Visceral Fat region (width of outside of abdominal cavity | 13886 | 0.043 | 13259 | | 0.043 | 260 | 0.043 | 349 | 0.043 | 105 | 0.043 | | 104 | 0.043 |
| Cross-sectional area (cm squared) of fat outside abdominal cavit | 13886 | 0.043 | 13259 | | 0.043 | 260 | 0.043 | 349 | 0.043 | 105 | 0.043 | | 104 | 0.043 |
| Cross-sectional area (cm squared) of all fat in test region | 13886 | 0.043 | 13259 | | 0.043 | 260 | 0.043 | 349 | 0.043 | 105 | 0.043 | | 104 | 0.043 |
| Fat mass in grams (Android + Gynoid regions) | 13886 | 0.043 | 13259 | | 0.043 | 260 | 0.043 | 349 | 0.043 | 105 | 0.043 | | 104 | 0.043 |
| Lean mass in grams (Android + Gynoid regions). | 13886 | 0.043 | 13259 | | 0.043 | 260 | 0.043 | 349 | 0.043 | 105 | 0.043 | | 104 | 0.043 |
| Fat + Lean mass in grams (Android + Gynoid regions) | 13886 | 0.043 | 13259 | | 0.043 | 260 | 0.043 | 349 | 0.043 | 105 | 0.043 | | 104 | 0.043 |
| Percent Fat (Android + Gynoid regions) | 13886 | 0.043 | 13259 | | 0.043 | 260 | 0.043 | 349 | 0.043 | 105 | 0.043 | | 104 | 0.043 |
| Cross-sectional area (cm squared) of fat inside abdominal cavity | 13886 | 0.043 | 13259 | | 0.043 | 260 | 0.043 | 349 | 0.043 | 105 | 0.043 | | 104 | 0.043 |
| Fat Tissue in largest Visceral Fat region (grams) | 13886 | 0.043 | 13259 | | 0.043 | 260 | 0.043 | 349 | 0.043 | 105 | 0.043 | | 104 | 0.043 |
| Lean Tissue in largest Visceral Fat region (grams) | 13886 | 0.043 | 13259 | | 0.043 | 260 | 0.043 | 349 | 0.043 | 105 | 0.043 | | 104 | 0.043 |
| All Tissue in largest Visceral Fat region (grams) | 13886 | 0.043 | 13259 | | 0.043 | 260 | 0.043 | 349 | 0.043 | 105 | 0.043 | | 104 | 0.043 |
| Percent fat in largest Visceral Fat region | 13886 | 0.043 | 13259 | | 0.043 | 260 | 0.043 | 349 | 0.043 | 105 | 0.043 | | 104 | 0.043 |
| Fat Tissue in inner-most Visceral Fat region (grams) | 13886 | 0.043 | 13259 | | 0.043 | 260 | 0.043 | 349 | 0.043 | 105 | 0.043 | | 104 | 0.043 |
| Lean Tissue in inner-most Visceral Fat region (grams) | 13886 | 0.043 | 13259 | | 0.043 | 260 | 0.043 | 349 | 0.043 | 105 | 0.043 | | 104 | 0.043 |
| All Tissue in inner-most Visceral Fat region (grams) | 13886 | 0.043 | 13259 | | 0.043 | 260 | 0.043 | 349 | 0.043 | 105 | 0.043 | | 104 | 0.043 |
| Percent fat in inner-most Visceral Fat region | 13886 | 0.043 | 13259 | | 0.043 | 260 | 0.043 | 349 | 0.043 | 105 | 0.043 | | 104 | 0.043 |
| Mass of fat (grams) inside abdominal cavity (in test region) | 13886 | 0.043 | 13259 | | 0.043 | 260 | 0.043 | 349 | 0.043 | 105 | 0.043 | | 104 | 0.043 |
| Fat Tissue in middle Visceral Fat region (grams) | 13886 | 0.043 | 13259 | | 0.043 | 260 | 0.043 | 349 | 0.043 | 105 | 0.043 | | 104 | 0.043 |
| Lean Tissue in middle Visceral Fat region (grams) | 13886 | 0.043 | 13259 | | 0.043 | 260 | 0.043 | 349 | 0.043 | 105 | 0.043 | | 104 | 0.043 |
| All Tissue in middle Visceral Fat region (grams) | 13886 | 0.043 | 13259 | | 0.043 | 260 | 0.043 | 349 | 0.043 | 105 | 0.043 | | 104 | 0.043 |
| Percent fat in middle Visceral Fat region | 13886 | 0.043 | 13259 | | 0.043 | 260 | 0.043 | 349 | 0.043 | 105 | 0.043 | | 104 | 0.043 |
| Volume of fat (cc) inside abdominal cavity (in test region) | 13886 | 0.043 | 13259 | | 0.043 | 260 | 0.043 | 349 | 0.043 | 105 | 0.043 | | 104 | 0.043 |
| Pure lean mass (without bone) in limbs | 13886 | 0.043 | 13259 | | 0.043 | 260 | 0.043 | 349 | 0.043 | 105 | 0.043 | | 104 | 0.043 |
| Pure lean mass (without bone) in head | 13886 | 0.043 | 13259 | | 0.043 | 260 | 0.043 | 349 | 0.043 | 105 | 0.043 | | 104 | 0.043 |
| Pure lean mass (without bone) in left arm | 13886 | 0.043 | 13259 | | 0.043 | 260 | 0.043 | 349 | 0.043 | 105 | 0.043 | | 104 | 0.043 |
| Pure lean mass (without bone) in left leg | 13886 | 0.043 | 13259 | | 0.043 | 260 | 0.043 | 349 | 0.043 | 105 | 0.043 | | 104 | 0.043 |
| Pure lean mass (without bone) in right arm | 13886 | 0.043 | 13259 | | 0.043 | 260 | 0.043 | 349 | 0.043 | 105 | 0.043 | | 104 | 0.043 |
| Pure lean mass (without bone) in right leg | 13886 | 0.043 | 13259 | | 0.043 | 260 | 0.043 | 349 | 0.043 | 105 | 0.043 | | 104 | 0.043 |
| Total pure lean mass (without bone) excluding head region | 13886 | 0.043 | 13259 | | 0.043 | 260 | 0.043 | 349 | 0.043 | 105 | 0.043 | | 104 | 0.043 |
| Pure lean mass (without bone) in trunk | 13886 | 0.043 | 13259 | | 0.043 | 260 | 0.043 | 349 | 0.043 | 105 | 0.043 | | 104 | 0.043 |
| Length of Left Hip Axis in cm | 11134 | 0.233 | 10711 | | 0.233 | 200 | 0.233 | 270 | 0.233 | 74 | 0.233 | | 75 | 0.233 |
| Femur Shaft Average Cortical Thickness (cm) - left hip | 11134 | 0.233 | 10711 | | 0.233 | 200 | 0.233 | 270 | 0.233 | 74 | 0.233 | | 75 | 0.233 |
| Femur Shaft Bone Mineral Density (g/cm2) - left hip | 11134 | 0.233 | 10711 | | 0.233 | 200 | 0.233 | 270 | 0.233 | 74 | 0.233 | | 75 | 0.233 |
| Femur Shaft Buckling Ratio (dimensionless) - left hip | 11134 | 0.233 | 10711 | | 0.233 | 200 | 0.233 | 270 | 0.233 | 74 | 0.233 | | 75 | 0.233 |
| Femur Shaft Center of Mass Position (dimensionless) - left hip | 11134 | 0.233 | 10711 | | 0.233 | 200 | 0.233 | 270 | 0.233 | 74 | 0.233 | | 75 | 0.233 |
| Femur Shaft Cross Sectional Area (cm2) - left hip | 11134 | 0.233 | 10711 | | 0.233 | 200 | 0.233 | 270 | 0.233 | 74 | 0.233 | | 75 | 0.233 |
| Femur Shaft Cross Sectional Moment of Inertia (cm4) - left hip | 11134 | 0.233 | 10711 | | 0.233 | 200 | 0.233 | 270 | 0.233 | 74 | 0.233 | | 75 | 0.233 |
| Femur Shaft Endocortical Diameter (cm) - left hip | 11134 | 0.233 | 10711 | | 0.233 | 200 | 0.233 | 270 | 0.233 | 74 | 0.233 | | 75 | 0.233 |
| Femur Shaft Profile Center Distance (cm) - left hip | 11134 | 0.233 | 10711 | | 0.233 | 200 | 0.233 | 270 | 0.233 | 74 | 0.233 | | 75 | 0.233 |
| Femur Shaft Section Modulus (cm3) - left hip | 11134 | 0.233 | 10711 | | 0.233 | 200 | 0.233 | 270 | 0.233 | 74 | 0.233 | | 75 | 0.233 |
| Femur Shaft Subperiosteal Width (cm) - left hip | 11134 | 0.233 | 10711 | | 0.233 | 200 | 0.233 | 270 | 0.233 | 74 | 0.233 | | 75 | 0.233 |
| Area for Total Bone analysis for all included regions - left hip | 11134 | 0.233 | 10711 | | 0.233 | 200 | 0.233 | 270 | 0.233 | 74 | 0.233 | | 75 | 0.233 |
| BMC for Total Bone analysis for all included regions - left hip | 11134 | 0.233 | 10711 | | 0.233 | 200 | 0.233 | 270 | 0.233 | 74 | 0.233 | | 75 | 0.233 |
| BMD for Total Bone analysis for all included regions - left hip | 11134 | 0.233 | 10711 | | 0.233 | 200 | 0.233 | 270 | 0.233 | 74 | 0.233 | | 75 | 0.233 |
| Area for inter -Trochanter Bone analysis - left hip | 11134 | 0.233 | 10711 | | 0.233 | 200 | 0.233 | 270 | 0.233 | 74 | 0.233 | | 75 | 0.233 |
| BMC for inter -Trochanter Bone analysis - left hip | 11134 | 0.233 | 10711 | | 0.233 | 200 | 0.233 | 270 | 0.233 | 74 | 0.233 | | 75 | 0.233 |
| BMD for inter -Trochanter Bone analysis - left hip | 11134 | 0.233 | 10711 | | 0.233 | 200 | 0.233 | 270 | 0.233 | 74 | 0.233 | | 75 | 0.233 |
| Intertrochanter Average Cortical Thickness (cm) - left hip | 11134 | 0.233 | 10711 | | 0.233 | 200 | 0.233 | 270 | 0.233 | 74 | 0.233 | | 75 | 0.233 |
| Intertrochanter Bone Mineral Density (g/cm2) - left hip | 11134 | 0.233 | 10711 | | 0.233 | 200 | 0.233 | 270 | 0.233 | 74 | 0.233 | | 75 | 0.233 |
| Intertrochanter Buckling Ratio (dimensionless) - left hip | 11134 | 0.233 | 10711 | | 0.233 | 200 | 0.233 | 270 | 0.233 | 74 | 0.233 | | 75 | 0.233 |
| Intertrochanter Center of Mass Position (dimensionless) - left hip | 11134 | 0.233 | 10711 | | 0.233 | 200 | 0.233 | 270 | 0.233 | 74 | 0.233 | | 75 | 0.233 |
| Intertrochanter Cross Sectional Area (cm2) - left hip | 11134 | 0.233 | 10711 | | 0.233 | 200 | 0.233 | 270 | 0.233 | 74 | 0.233 | | 75 | 0.233 |
| Intertrochanter Cross Sectional Moment of Inertia (cm4) - left hip | 11134 | 0.233 | 10711 | | 0.233 | 200 | 0.233 | 270 | 0.233 | 74 | 0.233 | | 75 | 0.233 |
| Intertrochanter Endocortical Diameter (cm) - left hip | 11134 | 0.233 | 10711 | | 0.233 | 200 | 0.233 | 270 | 0.233 | 74 | 0.233 | | 75 | 0.233 |
| Intertrochanter Profile Center Distance (cm) - left hip | 11134 | 0.233 | 10711 | | 0.233 | 200 | 0.233 | 270 | 0.233 | 74 | 0.233 | | 75 | 0.233 |
| Intertrochanter Section Modulus (cm3) - left hip | 11134 | 0.233 | 10711 | | 0.233 | 200 | 0.233 | 270 | 0.233 | 74 | 0.233 | | 75 | 0.233 |
| Intertrochanter Subperiosteal Width (cm) - left hip | 11134 | 0.233 | 10711 | | 0.233 | 200 | 0.233 | 270 | 0.233 | 74 | 0.233 | | 75 | 0.233 |
| Area for Neck Bone analysis - left hip | 11134 | 0.233 | 10711 | | 0.233 | 200 | 0.233 | 270 | 0.233 | 74 | 0.233 | | 75 | 0.233 |
| BMC for Neck Bone analysis - left hip | 11134 | 0.233 | 10711 | | 0.233 | 200 | 0.233 | 270 | 0.233 | 74 | 0.233 | | 75 | 0.233 |
| BMD for Neck Bone analysis - left hip | 11134 | 0.233 | 10711 | | 0.233 | 200 | 0.233 | 270 | 0.233 | 74 | 0.233 | | 75 | 0.233 |
| Narrow Neck Average Cortical Thickness (cm) - left hip | 11134 | 0.233 | 10711 | | 0.233 | 200 | 0.233 | 270 | 0.233 | 74 | 0.233 | | 75 | 0.233 |
| Narrow Neck Bone Mineral Density (g/cm2) -Left hip | 11134 | 0.233 | 10711 | | 0.233 | 200 | 0.233 | 270 | 0.233 | 74 | 0.233 | | 75 | 0.233 |
| Narrow Neck Buckling Ratio (dimensionless) - left hip | 11134 | 0.233 | 10711 | | 0.233 | 200 | 0.233 | 270 | 0.233 | 74 | 0.233 | | 75 | 0.233 |
| Narrow Neck Center of Mass Position (dimensionless) - left hip | 11134 | 0.233 | 10711 | | 0.233 | 200 | 0.233 | 270 | 0.233 | 74 | 0.233 | | 75 | 0.233 |
| Narrow Neck Cross Sectional Area (cm2) - left hip | 11134 | 0.233 | 10711 | | 0.233 | 200 | 0.233 | 270 | 0.233 | 74 | 0.233 | | 75 | 0.233 |
| Narrow Neck Cross Sectional Moment of Inertia (cm4) - left hip | 11134 | 0.233 | 10711 | | 0.233 | 200 | 0.233 | 270 | 0.233 | 74 | 0.233 | | 75 | 0.233 |
| Narrow Neck Endocortical Diameter (cm) - left hip | 11134 | 0.233 | 10711 | | 0.233 | 200 | 0.233 | 270 | 0.233 | 74 | 0.233 | | 75 | 0.233 |
| Narrow Neck Profile Center Distance (cm) - left hip | 11134 | 0.233 | 10711 | | 0.233 | 200 | 0.233 | 270 | 0.233 | 74 | 0.233 | | 75 | 0.233 |
| Narrow Neck Section Modulus (cm3) - left hip | 11134 | 0.233 | 10711 | | 0.233 | 200 | 0.233 | 270 | 0.233 | 74 | 0.233 | | 75 | 0.233 |
| Narrow Neck Subperiosteal Width (cm) - left hip | 11134 | 0.233 | 10711 | | 0.233 | 200 | 0.233 | 270 | 0.233 | 74 | 0.233 | | 75 | 0.233 |
| Height of ROI in cm - Left hip | 11134 | 0.233 | 10711 | | 0.233 | 200 | 0.233 | 270 | 0.233 | 74 | 0.233 | | 75 | 0.233 |
| Width of ROI in cm - left hip | 11134 | 0.233 | 10711 | | 0.233 | 200 | 0.233 | 270 | 0.233 | 74 | 0.233 | | 75 | 0.233 |
| Angle formed by shaft center line and neck axis in degrees - left hip | 11134 | 0.233 | 10711 | | 0.233 | 200 | 0.233 | 270 | 0.233 | 74 | 0.233 | | 75 | 0.233 |
| Area for Trochanter Bone analysis - Left hip | 11134 | 0.233 | 10711 | | 0.233 | 200 | 0.233 | 270 | 0.233 | 74 | 0.233 | | 75 | 0.233 |
| BMC for Trochanter Bone analysis - Left hip | 11134 | 0.233 | 10711 | | 0.233 | 200 | 0.233 | 270 | 0.233 | 74 | 0.233 | | 75 | 0.233 |
| BMD for Trochanter Bone analysis - Left hip | 11134 | 0.233 | 10711 | | 0.233 | 200 | 0.233 | 270 | 0.233 | 74 | 0.233 | | 75 | 0.233 |
| Area for Ward‚Äôs Triangle Bone analysis - left hip | 11134 | 0.233 | 10711 | | 0.233 | 200 | 0.233 | 270 | 0.233 | 74 | 0.233 | | 75 | 0.233 |
| BMC for Ward‚Äôs Triangle Bone analysis - left hip | 11134 | 0.233 | 10711 | | 0.233 | 200 | 0.233 | 270 | 0.233 | 74 | 0.233 | | 75 | 0.233 |
| BMD for Ward‚Äôs Triangle Bone analysis - left hip | 11134 | 0.233 | 10711 | | 0.233 | 200 | 0.233 | 270 | 0.233 | 74 | 0.233 | | 75 | 0.233 |
| % fat in Android region / % fat in Gynoid region | 13886 | 0.043 | 13259 | | 0.043 | 260 | 0.043 | 349 | 0.043 | 105 | 0.043 | | 104 | 0.043 |
| % fat in Android region | 13886 | 0.043 | 13259 | | 0.043 | 260 | 0.043 | 349 | 0.043 | 105 | 0.043 | | 104 | 0.043 |
| Lean mass in limbs divided by height squared (kg/m2) | 13886 | 0.043 | 13259 | | 0.043 | 260 | 0.043 | 349 | 0.043 | 105 | 0.043 | | 104 | 0.043 |
| Lean mass without bone in limbs, divided by height squared (kg/m2) | 13886 | 0.043 | 13259 | | 0.043 | 260 | 0.043 | 349 | 0.043 | 105 | 0.043 | | 104 | 0.043 |
| Total body mass divided by height squared (kg/m2) | 13886 | 0.043 | 13259 | | 0.043 | 260 | 0.043 | 349 | 0.043 | 105 | 0.043 | | 104 | 0.043 |
| Total fat mass divided by height squared (kg/m2) | 13886 | 0.043 | 13259 | | 0.043 | 260 | 0.043 | 349 | 0.043 | 105 | 0.043 | | 104 | 0.043 |
| % fat in Trunk / % fat in legs | 13886 | 0.043 | 13259 | | 0.043 | 260 | 0.043 | 349 | 0.043 | 105 | 0.043 | | 104 | 0.043 |
| % fat in Gynoid region | 13886 | 0.043 | 13259 | | 0.043 | 260 | 0.043 | 349 | 0.043 | 105 | 0.043 | | 104 | 0.043 |
| Total lean mass divided by height squared (kg/m2) | 13886 | 0.043 | 13259 | | 0.043 | 260 | 0.043 | 349 | 0.043 | 105 | 0.043 | | 104 | 0.043 |
| Total lean mass without bone, divided by height squared (kg/m2) | 13886 | 0.043 | 13259 | | 0.043 | 260 | 0.043 | 349 | 0.043 | 105 | 0.043 | | 104 | 0.043 |
| Trunk fat / Limb fat | 13886 | 0.043 | 13259 | | 0.043 | 260 | 0.043 | 349 | 0.043 | 105 | 0.043 | | 104 | 0.043 |
| Total fat mass in whole body | 13886 | 0.043 | 13259 | | 0.043 | 260 | 0.043 | 349 | 0.043 | 105 | 0.043 | | 104 | 0.043 |
| Total lean mass in whole body | 13886 | 0.043 | 13259 | | 0.043 | 260 | 0.043 | 349 | 0.043 | 105 | 0.043 | | 104 | 0.043 |
| % of total body mass that is fat | 13886 | 0.043 | 13259 | | 0.043 | 260 | 0.043 | 349 | 0.043 | 105 | 0.043 | | 104 | 0.043 |
| Total lean mass without bone in whole body (in grams) | 13886 | 0.043 | 13259 | | 0.043 | 260 | 0.043 | 349 | 0.043 | 105 | 0.043 | | 104 | 0.043 |
| Grams of Fat tissue in Head region | 13886 | 0.043 | 13259 | | 0.043 | 260 | 0.043 | 349 | 0.043 | 105 | 0.043 | | 104 | 0.043 |
| Grams of Lean tissue in Head region | 13886 | 0.043 | 13259 | | 0.043 | 260 | 0.043 | 349 | 0.043 | 105 | 0.043 | | 104 | 0.043 |
| Mass in grams in Head region | 13886 | 0.043 | 13259 | | 0.043 | 260 | 0.043 | 349 | 0.043 | 105 | 0.043 | | 104 | 0.043 |
| Percentage of fat tissue to total tissue mass in Head region | 13886 | 0.043 | 13259 | | 0.043 | 260 | 0.043 | 349 | 0.043 | 105 | 0.043 | | 104 | 0.043 |
| Grams of Fat tissue in Left arm region | 13886 | 0.043 | 13259 | | 0.043 | 260 | 0.043 | 349 | 0.043 | 105 | 0.043 | | 104 | 0.043 |
| Grams of Lean tissue in Left arm region | 13886 | 0.043 | 13259 | | 0.043 | 260 | 0.043 | 349 | 0.043 | 105 | 0.043 | | 104 | 0.043 |
| Mass in grams in Left arm region | 13886 | 0.043 | 13259 | | 0.043 | 260 | 0.043 | 349 | 0.043 | 105 | 0.043 | | 104 | 0.043 |
| Percentage of fat tissue to total tissue mass in Left arm region | 13886 | 0.043 | 13259 | | 0.043 | 260 | 0.043 | 349 | 0.043 | 105 | 0.043 | | 104 | 0.043 |
| Grams of Fat tissue in Left leg region | 13886 | 0.043 | 13259 | | 0.043 | 260 | 0.043 | 349 | 0.043 | 105 | 0.043 | | 104 | 0.043 |
| Grams of Lean tissue in Left leg region | 13886 | 0.043 | 13259 | | 0.043 | 260 | 0.043 | 349 | 0.043 | 105 | 0.043 | | 104 | 0.043 |
| Mass in grams in Left leg region | 13886 | 0.043 | 13259 | | 0.043 | 260 | 0.043 | 349 | 0.043 | 105 | 0.043 | | 104 | 0.043 |
| Percentage of fat tissue to total tissue mass in Left leg region | 13886 | 0.043 | 13259 | | 0.043 | 260 | 0.043 | 349 | 0.043 | 105 | 0.043 | | 104 | 0.043 |
| Grams of Fat tissue in Right arm region | 13886 | 0.043 | 13259 | | 0.043 | 260 | 0.043 | 349 | 0.043 | 105 | 0.043 | | 104 | 0.043 |
| Grams of Lean tissue in Right arm region | 13886 | 0.043 | 13259 | | 0.043 | 260 | 0.043 | 349 | 0.043 | 105 | 0.043 | | 104 | 0.043 |
| Mass in grams in Right arm region | 13886 | 0.043 | 13259 | | 0.043 | 260 | 0.043 | 349 | 0.043 | 105 | 0.043 | | 104 | 0.043 |
| Percentage of fat tissue to total tissue mass in Right arm region | 13886 | 0.043 | 13259 | | 0.043 | 260 | 0.043 | 349 | 0.043 | 105 | 0.043 | | 104 | 0.043 |
| Grams of Fat tissue in Right leg region | 13886 | 0.043 | 13259 | | 0.043 | 260 | 0.043 | 349 | 0.043 | 105 | 0.043 | | 104 | 0.043 |
| Grams of Lean tissue in Right leg region | 13886 | 0.043 | 13259 | | 0.043 | 260 | 0.043 | 349 | 0.043 | 105 | 0.043 | | 104 | 0.043 |
| Mass in grams in Right leg region | 13886 | 0.043 | 13259 | | 0.043 | 260 | 0.043 | 349 | 0.043 | 105 | 0.043 | | 104 | 0.043 |
| Percentage of fat tissue to total tissue mass in Right leg region | 13886 | 0.043 | 13259 | | 0.043 | 260 | 0.043 | 349 | 0.043 | 105 | 0.043 | | 104 | 0.043 |
| Grams of Fat tissue in Summary | 13886 | 0.043 | 13259 | | 0.043 | 260 | 0.043 | 349 | 0.043 | 105 | 0.043 | | 104 | 0.043 |
| Grams of Lean tissue in Summary | 13886 | 0.043 | 13259 | | 0.043 | 260 | 0.043 | 349 | 0.043 | 105 | 0.043 | | 104 | 0.043 |
| Mass in grams in Summary | 13886 | 0.043 | 13259 | | 0.043 | 260 | 0.043 | 349 | 0.043 | 105 | 0.043 | | 104 | 0.043 |
| Percentage of fat tissue to total tissue mass in Summary | 13886 | 0.043 | 13259 | | 0.043 | 260 | 0.043 | 349 | 0.043 | 105 | 0.043 | | 104 | 0.043 |
| Grams of Fat tissue in Trunk region | 13886 | 0.043 | 13259 | | 0.043 | 260 | 0.043 | 349 | 0.043 | 105 | 0.043 | | 104 | 0.043 |
| Grams of Lean tissue in Trunk region | 13886 | 0.043 | 13259 | | 0.043 | 260 | 0.043 | 349 | 0.043 | 105 | 0.043 | | 104 | 0.043 |
| Mass in grams in Trunk region | 13886 | 0.043 | 13259 | | 0.043 | 260 | 0.043 | 349 | 0.043 | 105 | 0.043 | | 104 | 0.043 |
| Percentage of fat tissue to total tissue mass in Trunk region | 13886 | 0.043 | 13259 | | 0.043 | 260 | 0.043 | 349 | 0.043 | 105 | 0.043 | | 104 | 0.043 |
| Grams of Fat tissue in all included regions | 13886 | 0.043 | 13259 | | 0.043 | 260 | 0.043 | 349 | 0.043 | 105 | 0.043 | | 104 | 0.043 |
| Grams of Lean tissue in all included regions | 13886 | 0.043 | 13259 | | 0.043 | 260 | 0.043 | 349 | 0.043 | 105 | 0.043 | | 104 | 0.043 |
| Mass in grams in all included regions | 13886 | 0.043 | 13259 | | 0.043 | 260 | 0.043 | 349 | 0.043 | 105 | 0.043 | | 104 | 0.043 |
| Percentage of fat tissue to total tissue mass in all included regions | 13886 | 0.043 | 13259 | | 0.043 | 260 | 0.043 | 349 | 0.043 | 105 | 0.043 | | 104 | 0.043 |
| Bone area for the Head region Bone analysis | 13886 | 0.043 | 13259 | | 0.043 | 260 | 0.043 | 349 | 0.043 | 105 | 0.043 | | 104 | 0.043 |
| Bone mineral content for the Head region | 13886 | 0.043 | 13259 | | 0.043 | 260 | 0.043 | 349 | 0.043 | 105 | 0.043 | | 104 | 0.043 |
| Bone mineral density for the Head region | 13886 | 0.043 | 13259 | | 0.043 | 260 | 0.043 | 349 | 0.043 | 105 | 0.043 | | 104 | 0.043 |
| Bone area for the Left Arm region | 13886 | 0.043 | 13259 | | 0.043 | 260 | 0.043 | 349 | 0.043 | 105 | 0.043 | | 104 | 0.043 |
| Bone mineral content for the Left Arm region | 13886 | 0.043 | 13259 | | 0.043 | 260 | 0.043 | 349 | 0.043 | 105 | 0.043 | | 104 | 0.043 |
| Bone mineral density for the Left Arm region | 13886 | 0.043 | 13259 | | 0.043 | 260 | 0.043 | 349 | 0.043 | 105 | 0.043 | | 104 | 0.043 |
| Bone area for the Left Leg region | 13886 | 0.043 | 13259 | | 0.043 | 260 | 0.043 | 349 | 0.043 | 105 | 0.043 | | 104 | 0.043 |
| Bone mineral content for the Left Leg region | 13886 | 0.043 | 13259 | | 0.043 | 260 | 0.043 | 349 | 0.043 | 105 | 0.043 | | 104 | 0.043 |
| Bone mineral density for the Left Leg region | 13886 | 0.043 | 13259 | | 0.043 | 260 | 0.043 | 349 | 0.043 | 105 | 0.043 | | 104 | 0.043 |
| Bone area for the Left Rib region | 13886 | 0.043 | 13259 | | 0.043 | 260 | 0.043 | 349 | 0.043 | 105 | 0.043 | | 104 | 0.043 |
| Bone mineral content for the Left Rib region | 13886 | 0.043 | 13259 | | 0.043 | 260 | 0.043 | 349 | 0.043 | 105 | 0.043 | | 104 | 0.043 |
| Bone mineral density for the Left Rib region | 13886 | 0.043 | 13259 | | 0.043 | 260 | 0.043 | 349 | 0.043 | 105 | 0.043 | | 104 | 0.043 |
| Bone area for the Lumbar Spine region | 13886 | 0.043 | 13259 | | 0.043 | 260 | 0.043 | 349 | 0.043 | 105 | 0.043 | | 104 | 0.043 |
| Bone mineral content for the Lumbar Spine region | 13886 | 0.043 | 13259 | | 0.043 | 260 | 0.043 | 349 | 0.043 | 105 | 0.043 | | 104 | 0.043 |
| Bone mineral density for the Lumbar Spine region | 13886 | 0.043 | 13259 | | 0.043 | 260 | 0.043 | 349 | 0.043 | 105 | 0.043 | | 104 | 0.043 |
| Bone area for the Pelvic region | 13886 | 0.043 | 13259 | | 0.043 | 260 | 0.043 | 349 | 0.043 | 105 | 0.043 | | 104 | 0.043 |
| Bone mineral content for the Pelvic region | 13886 | 0.043 | 13259 | | 0.043 | 260 | 0.043 | 349 | 0.043 | 105 | 0.043 | | 104 | 0.043 |
| Bone mineral density for the Pelvic region | 13886 | 0.043 | 13259 | | 0.043 | 260 | 0.043 | 349 | 0.043 | 105 | 0.043 | | 104 | 0.043 |
| Bone area for the Right Arm region | 13886 | 0.043 | 13259 | | 0.043 | 260 | 0.043 | 349 | 0.043 | 105 | 0.043 | | 104 | 0.043 |
| Bone mineral content for the Right Arm region | 13886 | 0.043 | 13259 | | 0.043 | 260 | 0.043 | 349 | 0.043 | 105 | 0.043 | | 104 | 0.043 |
| Bone mineral density for the Right Arm region | 13886 | 0.043 | 13259 | | 0.043 | 260 | 0.043 | 349 | 0.043 | 105 | 0.043 | | 104 | 0.043 |
| Bone area for the Right Leg region | 13886 | 0.043 | 13259 | | 0.043 | 260 | 0.043 | 349 | 0.043 | 105 | 0.043 | | 104 | 0.043 |
| Bone mineral content for the Right Leg region | 13886 | 0.043 | 13259 | | 0.043 | 260 | 0.043 | 349 | 0.043 | 105 | 0.043 | | 104 | 0.043 |
| Bone mineral density for the Right Leg region | 13886 | 0.043 | 13259 | | 0.043 | 260 | 0.043 | 349 | 0.043 | 105 | 0.043 | | 104 | 0.043 |
| Bone area for the Right Rib region | 13886 | 0.043 | 13259 | | 0.043 | 260 | 0.043 | 349 | 0.043 | 105 | 0.043 | | 104 | 0.043 |
| Bone mineral content for the Right Rib region | 13886 | 0.043 | 13259 | | 0.043 | 260 | 0.043 | 349 | 0.043 | 105 | 0.043 | | 104 | 0.043 |
| Bone mineral density for the Right Rib region | 13886 | 0.043 | 13259 | | 0.043 | 260 | 0.043 | 349 | 0.043 | 105 | 0.043 | | 104 | 0.043 |
| Total bone area excluding the head region | 13886 | 0.043 | 13259 | | 0.043 | 260 | 0.043 | 349 | 0.043 | 105 | 0.043 | | 104 | 0.043 |
| Total BMC excluding the head region | 13886 | 0.043 | 13259 | | 0.043 | 260 | 0.043 | 349 | 0.043 | 105 | 0.043 | | 104 | 0.043 |
| Total BMD excluding the head region | 13886 | 0.043 | 13259 | | 0.043 | 260 | 0.043 | 349 | 0.043 | 105 | 0.043 | | 104 | 0.043 |
| Bone area for the Thoracic region | 13886 | 0.043 | 13259 | | 0.043 | 260 | 0.043 | 349 | 0.043 | 105 | 0.043 | | 104 | 0.043 |
| Bone mineral content for the Thoracic region | 13886 | 0.043 | 13259 | | 0.043 | 260 | 0.043 | 349 | 0.043 | 105 | 0.043 | | 104 | 0.043 |
| Bone mineral density for the Thoracic region | 13886 | 0.043 | 13259 | | 0.043 | 260 | 0.043 | 349 | 0.043 | 105 | 0.043 | | 104 | 0.043 |
| Total body area | 13886 | 0.043 | 13259 | | 0.043 | 260 | 0.043 | 349 | 0.043 | 105 | 0.043 | | 104 | 0.043 |
| Total BMC | 13886 | 0.043 | 13259 | | 0.043 | 260 | 0.043 | 349 | 0.043 | 105 | 0.043 | | 104 | 0.043 |
| Total BMD | 13886 | 0.043 | 13259 | | 0.043 | 260 | 0.043 | 349 | 0.043 | 105 | 0.043 | | 104 | 0.043 |
| P Axis | 13773 | 0.065 | 12741 | | 0.065 | 261 | 0.065 | 342 | 0.065 | 104 | 0.065 | | 101 | 0.065 |
| P Duration | 13763 | 0.067 | 12697 | | 0.067 | 261 | 0.067 | 342 | 0.067 | 103 | 0.067 | | 101 | 0.067 |
| P Offset | 14047 | 0.044 | 13072 | | 0.044 | 263 | 0.044 | 346 | 0.044 | 107 | 0.044 | | 107 | 0.044 |
| P Onset | 14047 | 0.044 | 13072 | | 0.044 | 263 | 0.044 | 346 | 0.044 | 107 | 0.044 | | 107 | 0.044 |
| PP Interval | 14252 | 0.020 | 13556 | | 0.020 | 265 | 0.020 | 352 | 0.020 | 108 | 0.020 | | 108 | 0.020 |
| PQ Interval | 13753 | 0.068 | 12687 | | 0.068 | 261 | 0.068 | 341 | 0.068 | 104 | 0.068 | | 99 | 0.068 |
| Q Offset | 14252 | 0.020 | 13556 | | 0.020 | 265 | 0.020 | 352 | 0.020 | 108 | 0.020 | | 108 | 0.020 |
| Q Onset | 14252 | 0.020 | 13556 | | 0.020 | 265 | 0.020 | 352 | 0.020 | 108 | 0.020 | | 108 | 0.020 |
| QRS Duration | 14255 | 0.020 | 13557 | | 0.020 | 265 | 0.020 | 352 | 0.020 | 108 | 0.020 | | 108 | 0.020 |
| QRS Number | 14252 | 0.020 | 13556 | | 0.020 | 265 | 0.020 | 352 | 0.020 | 108 | 0.020 | | 108 | 0.020 |
| QTC Interval | 14255 | 0.020 | 13557 | | 0.020 | 265 | 0.020 | 352 | 0.020 | 108 | 0.020 | | 108 | 0.020 |
| QT Interval | 14255 | 0.020 | 13557 | | 0.020 | 265 | 0.020 | 352 | 0.020 | 108 | 0.020 | | 108 | 0.020 |
| R Axis | 14251 | 0.020 | 13550 | | 0.020 | 265 | 0.020 | 352 | 0.020 | 108 | 0.020 | | 108 | 0.020 |
| RR Interval | 14232 | 0.021 | 13543 | | 0.021 | 265 | 0.021 | 351 | 0.021 | 108 | 0.021 | | 108 | 0.021 |
| T Axis | 14251 | 0.020 | 13556 | | 0.020 | 265 | 0.020 | 352 | 0.020 | 108 | 0.020 | | 108 | 0.020 |
| T Offset | 14252 | 0.020 | 13556 | | 0.020 | 265 | 0.020 | 352 | 0.020 | 108 | 0.020 | | 108 | 0.020 |
| Ventricular Rate | 14255 | 0.020 | 13557 | | 0.020 | 265 | 0.020 | 352 | 0.020 | 108 | 0.020 | | 108 | 0.020 |
| Average height in m | 14479 | 0.003 | 13798 | | 0.003 | 267 | 0.003 | 360 | 0.003 | 110 | 0.003 | | 110 | 0.003 |
| Body Mass Index | 14459 | 0.004 | 13784 | | 0.004 | 267 | 0.004 | 360 | 0.004 | 110 | 0.004 | | 110 | 0.004 |
| Average weight in kg | 14461 | 0.004 | 13787 | | 0.004 | 267 | 0.004 | 360 | 0.004 | 110 | 0.004 | | 110 | 0.004 |
| Hips circumference in cm | 14418 | 0.008 | 13733 | | 0.008 | 267 | 0.008 | 356 | 0.008 | 109 | 0.008 | | 110 | 0.008 |
| Waist to hip ratio | 14418 | 0.008 | 13733 | | 0.008 | 267 | 0.008 | 356 | 0.008 | 109 | 0.008 | | 110 | 0.008 |
| Waist circumference in cm | 14418 | 0.008 | 13733 | | 0.008 | 267 | 0.008 | 356 | 0.008 | 109 | 0.008 | | 110 | 0.008 |

**Table S4. Health-related phenotypes that showed heteroskedasticity with a lower variance among the healthiest group that were detected only with one Instrument in self-reported white CLSA participants.**

| **Instrument*** | **Phenotype** |
| --- | --- |
| **I** |  |
|  | Ferritin, µg/L |
|  | Thyroid-Stimulating Hormone, mIU/L |
| **II** |  |
|  | Albumin, g/L |
|  | non HDL, mmol/L |
|  | Intertrochanter Cross Sectional Moment of Inertia (cm4) - left hip |
|  | Intertrochanter Section Modulus (cm3) - left hip |
|  | Narrow Neck Average Cortical Thickness (cm) - left hip |
|  | Narrow Neck Bone Mineral Density (g/cm2) -Left hip |
|  | Bone area for the Left Arm region |
|  | Bone mineral density for the Lumbar Spine region |
|  | Bone area for the Pelvic region |
|  | Total BMD |
|  | P Duration, ms |
|  | P Offset, ms |
|  | Q Onset, ms |
|  | QRS Number |
| **III** |  |
|  | Bone mineral content for the Right Arm region |
| **IV** |  |
|  | Hemoglobin, g/L |
|  | Mean corpuscular volume, fL |
|  | Percent fat in Gynoid region |
|  | Percent Fat (Android + Gynoid regions) |
|  | Femur Shaft Cross Sectional Area (cm2) - left hip |
|  | Femur Shaft Section Modulus (cm3) - left hip |
|  | Narrow Neck Cross Sectional Area (cm2) - left hip |
|  | % fat in Gynoid region |
|  | Percentage of fat tissue to total tissue mass in Summary |
|  | Percentage of fat tissue to total tissue mass in Trunk region |
|  | Bone area for the Left Rib region |
|  | Bone area for the Right Leg region |
|  | Bone area for the Right Rib region |
|  | Bone mineral content for the Thoracic region |
|  | Ventricular Rate, BPM |

* Instruments: I - the Frailty Index; II - the number of five diseases: cancer (except non-melanoma skin cancer), cardiovascular disease, major pulmonary disease, dementia, and diabetes; III - the number of other chronic conditions; IV - composite cognitive score; V- physical functioning

**Table S5.** **Health-related phenotypes that showed heteroskedasticity with a lower variance among the healthiest group that were detected only for one sex in self-reported white CLSA participants.**

| **Sex** | **Phenotype** |
| --- | --- |
| **Females** |  |
|  | Cholesterol, mmol/L |
|  | Ferritin, µg/L |
|  | Low-Density Lipoprotein, calculated, mmol/L |
|  | Mean corpuscular volume, fL |
|  | Monocytes (absolute number), 10^9^/L |
|  | non HDL, mmol/L |
|  | Triglycerides, mmol/L |
|  | Thyroid-Stimulating Hormone, mIU/L |
|  | Average pluse rate (excluding 1st reading) |
|  | Pure lean mass (without bone) in left arm |
|  | Femur Shaft Cross Sectional Area (cm2) - left hip |
|  | Intertrochanter Cross Sectional Area (cm2) - left hip |
|  | Intertrochanter Cross Sectional Moment of Inertia (cm4) - left hip |
|  | Intertrochanter Section Modulus (cm3) - left hip |
|  | Narrow Neck Average Cortical Thickness (cm) - left hip |
|  | Narrow Neck Bone Mineral Density (g/cm2) -Left hip |
|  | Narrow Neck Cross Sectional Area (cm2) - left hip |
|  | Trunk fat / Limb fat |
|  | Grams of Lean tissue in Left arm region |
|  | Bone area for the Left Arm region |
|  | Bone mineral density for the Lumbar Spine region |
|  | Bone area for the Pelvic region |
|  | Bone mineral content for the Pelvic region |
|  | Bone area for the Right Arm region |
|  | Bone mineral content for the Right Arm region |
|  | Total BMC |
|  | Total BMD |
|  | QRS Number |
|  | Ventricular Rate, BPM |
|  |  |
| **Males** |  |
|  | Hemoglobin, g/L |
|  | Percent fat in Gynoid region |
|  | Percent Fat (Android + Gynoid regions) |
|  | Femur Shaft Section Modulus (cm3) - left hip |
|  | % fat in Gynoid region |
|  | % of total body mass that is fat |
|  | Percentage of fat tissue to total tissue mass in Left arm region |
|  | Percentage of fat tissue to total tissue mass in Left leg region |
|  | Percentage of fat tissue to total tissue mass in Right arm region |
|  | Percentage of fat tissue to total tissue mass in Right leg region |
|  | Percentage of fat tissue to total tissue mass in Summary |
|  | Percentage of fat tissue to total tissue mass in Trunk region |
|  | Percentage of fat tissue to total tissue mass in all included regions |
|  | Bone area for the Left Rib region |
|  | Bone area for the Right Leg region |
|  | Bone area for the Right Rib region |
|  | Bone mineral content for the Thoracic region |
|  | P Duration, ms |
